# Development of bicistronic expression system for the enhanced and reliable production of recombinant proteins in *Leuconostoc citreum*

**DOI:** 10.1038/s41598-018-27091-z

**Published:** 2018-06-11

**Authors:** Seung Hoon Jang, Ji Won Cha, Nam Soo Han, Ki Jun Jeong

**Affiliations:** 1 Department of Chemical and Biomolecular Engineering (BK21 Program), KAIST, 291 Daehak-ro, Yuseong-gu, Daejeon, 34141 Republic of Korea; 2 Brain Korea 21 Center for Bio-Resource Development, Division of Animal, Horticultural and Food Sciences, Chungbuk National University, Cheongju, 28644 Republic of Korea; 3 KAIST Institute for the BioCentury, 291 Daehak-ro, Yuseong-gu, Daejeon, 34141 Republic of Korea

## Abstract

The lactic acid bacteria (LAB) *Leuconostoc citreum* are non-sporulating hetero-fermentative bacteria that play an important role in the fermented food industry. In this study, for the enhanced and reliable production of recombinant proteins in *L*. *citreum*, we developed a bicistronic design (BCD) expression system which includes a short leader peptide (1^st^ cistron) followed by target genes (2^nd^ cistron) under the control of a single promoter. Using superfolder green fluorescent protein (sfGFP) as a reporter, the functionality of BCD in *L*. *citreum* was verified. Further, to improve the expression in BCD, we tried to engineer a Shine-Dalgarno sequence (SD2) for the 2^nd^ cistron and a promoter by FACS screening of random libraries, and both strong SD2 (eSD2) and promoter (P_710V4_) were successfully isolated. The usefulness of the engineered BCD with P_710V4_ and eSD2 was further validated using three model proteins—glutathione-s-transferase, human growth hormone, and α-amylase. All examined proteins were successfully produced with levels highly increased compared with those in the original BCD as well as the monocistronic design (MCD) expression system.

## Introduction

The lactic acid bacteria (LAB) *Leuconostoc* sp. are gram-positive, non-sporulating, and food grade bacteria^1^. Their remarkable fermentation capability plays a pivotal role in the fermentation of milk, vegetable, meat, wine, and other dairy products, and can be used for food safety and health benefits^2,3^. The aromatic compounds produced by *Leuconostoc* sp. are used as flavor-enhancers in food products and dextran is used in food additives or industrial products such as Sephadex^4,5^. Additionally, *Leuconostoc* sp. produce d-lactate at a relatively high optical purity and titer^6^. Health-promoting molecules such as vitamins and bioactive antimicrobial peptides can also be produced by *Leuconostoc* sp.^7,8^. Similar to other LAB such as *Lactobacillus* sp., *Leuconostoc* sp. are also a generally recognized as safe (GRAS) strain; these strains are garnering great attention as a host for the production of recombinant proteins (particularly protein therapeutics) because of its enhanced protein secretion ability^9,10^. However, due to the limitation of genetic tools for gene expression, there has not been much progress in the engineering of *Leuconostoc* sp. as a potential host for the recombinant protein production.

Over the past decades, many studies have concentrated on the development of plasmids for use in LAB^2,10–13^. Recently, we also developed a high-copy-number plasmid that occurs at about 60 copies per cell, and using this, overproduction of various recombinant proteins could be achieved in *Leuconostoc citreum*^14^. Although increased plasmid copy number is a good strategy for high-level gene expression, more genetic factors, including promoter strength, coding sequence (codon usage), untranslated region (UTR)/translation initiation region (TIR) sequence, and transcription terminator, can critically affect the expression level. Hence these factors should be taken into account for more efficient and stronger expression of any gene of interest (GOI)^15^. The promoter is a primary factor in the construction of expression system, because expression levels of GOIs are highly dependent on promoter strength and can be easily controlled by employing different-strength promoters—strong and weak promoter for higher and lower gene expression, respectively^16–18^. However, it is also well known that gene expression levels are not always correlated with promoter strength because of the influence of other genetic elements such as the 5′ UTR and TIR in mRNA trnascript^19^. The UTR/TIR sequences affect the mRNA secondary structure formation and mRNA stability^20,21^. Owing to unfavorable secondary structures in the UTR/TIR regions, low or no expression of genes were noted in the general monocistronic design (MCD) system although the expression of target genes was under the control of strong promoters. To overcome this problem and achieve a reliable gene expression, it is necessary to optimize the sequence of UTR/TIR region for each target gene, which can be time-consuming. For the reliable and precise control of gene expression, a bicistronic design (BCD) can be considered, instead of MCD, in which the Shine-Dalgarno (SD) sequence for GOI (2^nd^ cistron) is entirely embedded in the 3′-end of the short (below 20 amino acids) coding sequences (1^st^ cistron) under a single promoter. In this architecture, the translation of the GOI results primarily from SD1-directed ribosomes that recognize and reinitiate translation via a second SD (SD2), and the optimized sequence of 1^st^ cistron is favorable for transcription/translation initiation, ensuring high expression of the target gene (2^nd^ cistron) by translational coupling^22,23^.

Recently, Mutalik *et al*.^22^, using synthetic BCD and promoters, successfully demonstrated the precisely controlled and reliable gene expression in *Escherichia coli*. Using SD parts for expression of 2^nd^ cistron with different strengths, expression levels of target genes could be precisely controlled with high reliability, which can be a powerful tool in the engineering of host cell. More recently, Zhao *et al*.^24^, also developed BCD for the enhanced expression of recombinant genes in *Corynebacterium glutamicum*. From the proteome analysis of *C*. *glutamicum*, a few highly-expressed genes were isolated and the genetic parts of each gene including its promoter and N-terminal fragment (17 a.a.) were employed as the 1^st^ cistron in BCD of *C*. *glutamicum*. Using GFP as a reporter, the optimized BCD exhibited up to 790-fold increased expression in *C*. *glutamicum* compared with conventional MCD.

Here, we developed a BCD system for the enhanced and reliable gene expression in *L*. *citreum*. First, we constructed the BCD system for the expression of sfGFP gene as a reporter. Next, we aimed to optimize the BCD for the high-level expression of target genes. For this purpose, the promoter and the 2^nd^ SD in the BCD were sequentially randomized and highly-fluorescent clones which have optimal BCD were isolated by fluorescent activated cell sorting (FACS)-based high-throughput screening. Using the optimized BCD, the enhanced and reliable production of recombinant proteins in *L*. *citreum* was successfully demonstrated using three protein models—glutathione-S-transferase (GST), human growth hormone (hGH), and α-amylase from *Lactobacillus amylovorus*.

## Results

### Construction of the BCD system for sfGFP expression

**Figure 1:**
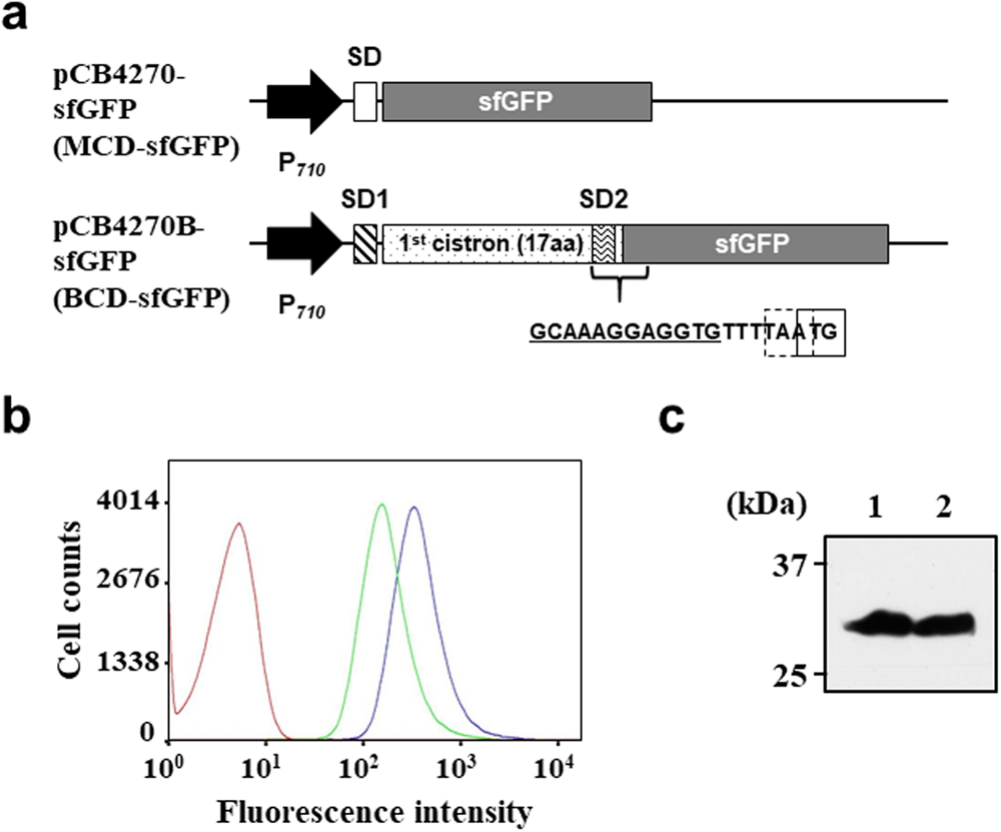
Development of BCD system in *L*. *citreum*. **(a)** Schematic diagram of two expression systems (BCD and MCD) for sfGFP gene expression. P_710_ indicates P_710_ constitutive promoter derived from *L*. *mesenteroides*. In BCD, SD2 and its junction with sfGFP gene are detailed and the sequence of SD2 is underlined. Dashed and solid boxes in the DNA sequences indicate the stop codon of 1^st^ cistron and start codon of 2^nd^ cistron, respectively. **(b)** Analysis of sfGFP fluorescence by FACS. In histogram of FACS analysis, pCB4270-sfGFP and pCB4270B-sfGFP are represented by blue and green, respectively. A red curve shows the negative control (pCB4270). **(c)** Analysis of sfGFP expression by western blotting. Protein samples were taken at 10 hr cultivation and total fractions were loaded. Lane 1, pCB4270-sfGFP; lane 2, pCB4270B-sfGFP. Uncropped blot is shown in Supplementary Figure S11.

In the BCD platform, the 2^nd^ cistron can be expressed by translational coupling with the 1^st^ cistron and the success depends on the efficiency of this coupling in the host^23,25,26^. In several bacterial hosts such as *E*. *coli* and *C*. *glutamicum*, the expression of the 2^nd^ cistron via translational coupling was successfully demonstrated^22,24^. Also, the development of translation coupling has been reported in a few LABs including *Lactococcus lactis* and *Lactobacillus sakei*^27,28^, but there has been no report in *Leuconostoc* sp. yet. First, to confirm whether a BCD system with translational coupling works or not in *L*. *citreum*, we constructed a BCD system using sfGFP as the 2^nd^ cistron and examined whether sfGFP gene in the BCD can be successfully expressed in *L*. *citreum*. In the BCD, the 1^st^ cistron comprising of the first SD sequence (SD1), the short leader peptide (1^st^ cistron) and the second SD sequence (SD2) for the 2^nd^ cistron was cloned between P_710_ promoter and sfGFP coding sequence (Fig. 1a). A sequence of the SD1 and 1^st^ cistronic gene encoding 17 a.a.-length leader peptide was derived from a BCD2 sequence which was previously developed in *E*. *coli*^22^. The sequence ‘GCAAAGGAGGTG’ that is complementary to the sequence of *Leuconostoc mesenteroides* 16S rRNA (CACCTCCTTTGC) was used as SD2 site instead of *L*. *citreum* 16S rRNA whose anti-SD sequence information has not been reported yet^29^. The SD2 is located in the 3′-end of the 1^st^ cistron and serves as a ribosome binding site for the translation of the 2^nd^ cistron. A 1-bp frame shift between the stop codon of the 1^st^ cistron and the start codon of the 2^nd^ cistron was designed by which the 2^nd^ cistron can be expressed under the same promoter by translational coupling (Fig. 1a).

To check the expression level of sfGFP gene in the BCD, *L*. *citreum* harboring pCB4270B-sfGFP was cultured and the fluorescent intensity (FI) was analyzed by flow cytometer. The plasmid pCB4270-sfGFP, which allows the expression of sfGFP in the MCD, was used as a control (Fig. 1a). In the BCD (pCB4270B-sfGFP), the FI of sfGFP was successfully observed although its value was relatively lower than that in the MCD system, but both systems exhibited sufficiently higher FIs than that of the negative control (no production of sfGFP) (Fig. 1b). The expression level and molecular weight of sfGFP produced in both systems were also confirmed by western blotting. As shown in Fig. 1c, a similar level of expression was observed in each expression system, and both proteins showed the same electrophoretic mobility in SDS-PAGE gel, reflecting similar molecular weights (approx. 26 kDa). Those results indicate that sfGFP present in the 2^nd^ cistron in the BCD could be accurately translated by translational coupling. Collectively, we concluded that the BCD system works effectively in *L*. *citreum*, and it was used for further engineering toward enhanced expression of the 2^nd^ cistron (target gene).

### Engineering of SD2 in the BCD

In bacterial hosts, the sequence of UTR including SD region significantly affects the gene expression levels, which also applies to the expression of the 2^nd^ cistron in the BCD. We carried out FACS screening of the SD2 random library to isolate an optimal SD2 sequence that can increase the expression of the 2^nd^ cistron in the BCD. The SD2 sequence (5′-GCAAAGGAGGTG-3′) in pCB4270B-sfGFP was randomized, preserving the 5′-nucleotide putative consensus core (underlined). The random library was first constructed in *E*. *coli* (2 × 10^7^ cells) and was subsequently moved to *L*. *citreum* by further transformation resulting in a library of 2 × 10^5^ cells. From the library, 25 clones were randomly picked, and sequencing their SD2 region revealed that each clone contained entirely different sequences in the SD2 region except for the ‘GGAGG’ consensus core (data not shown). In the first round of FACS screening, the population with high FI (top 5% of the total cells) was selectively sorted, and from the second round, top 1% of the total cells was selectively sorted. This procedure was repeated four more times with a total of six screening rounds, and the fluorescent cells were enriched more and more (Supplementary Fig. S1). After the sixth round, 20 individual clones were randomly selected and cultured in 96-deep well plates for the analysis of individual clones. Among the 20 clones, top eight clones which showed increased FIs than that of the cells harboring the original plasmid (pCB4270B-sfGFP) were selected (data not shown). Sequencing results revealed that all 8 clones had identical SD2 sequences with four mutations: G**G**AA**G***GGAGG***GT** (the mutated sequences are bold and underlined, and the consensus core sequences are italicized). The plasmid and SD2 of the isolated clone were named pCB4270BU-sfGFP and eSD2 (engineered SD2), respectively.

### Verification of the isolated eSD2 for gene expression

**Figure 2:**
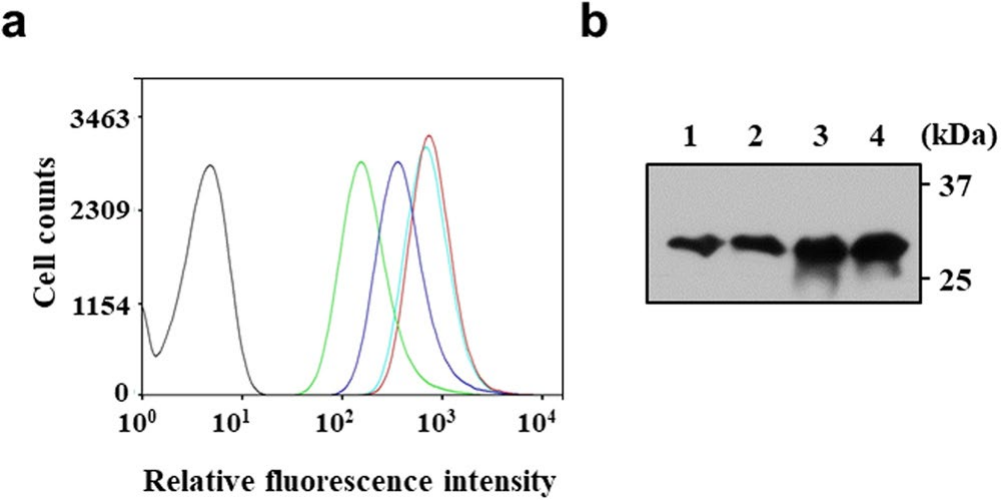
Verification of the isolated eSD2. **(a)** FACS analysis of sfGFP fluorescence. The histograms of pCB4270-sfGFP, pCB4270B-sfGFP, pCB4270BU-sfGFP and pCB4270BM-sfGFP are represented by blue, green, red and cyan, respectively. pCB4270 (black curve) was used as a negative control. **(b)** Western blotting of sfGFP expression. Lane 1, pCB4270-sfGFP; lane 2, pCB4270B-sfGFP; lane 3, pCB4270BU-sfGFP; lane 4, pCB4270BM-sfGFP. Uncropped blot is shown in Supplementary Figure S11.

The expression of sfGFP in the isolated clone (pCB4270BU-sfGFP) was analyzed by FACS and SDS-PAGE following by western blotting, and its levels were compared to that of the original BCD (pCB4270B-sfGFP) and MCD system (pCB4270-sfGFP). The isolated clone exhibited enhanced expression of sfGFP compared with those of the original BCD and MCD clones (Fig. 2a,b). In addition, to verify the contribution of the four mutations of eSD2 towards increased expression of sfGFP, the same mutations were introduced in the original SD2 in pCB4270B-sfGFP, yielding pCB4270BM-sfGFP. FACS analysis and western blotting clearly confirmed that the value of FI and expression level of sfGFP in pCB4270BM-sfGFP were nearly similar to that of the isolated pCB4270BU-sfGFP (Fig. 2a,b). This result also indicates the increase of gene expression in the isolated clone was a direct result of the mutations (eSD2) in the SD2 region and not due to any possible mutation in the plasmid or chromosomal DNA of the host. We considered two possibilities to explain the positive effect of eSD2 on gene expression: (i) increase in the transcript level, or (ii) increased mRNA stability. To examine these possibilities, we performed quantitative reverse transcription PCR (qRT-PCR) with mRNA purified from *L*. *citreum* in which transcription was stopped by the addition of rifampicin^30,31^. Comparative analysis of the mRNA levels and relative mRNA decay (original SD2 and eSD2) indicated lack of significant difference between the original and engineered BCD (Supplementary Fig. S2), implying that use of the eSD2 did not change the transcription levels and mRNA stability. We suppose that the increased gene expression may be a result of the enhanced translational efficiency with the eSD2 sequence after transcription^32–35^.

### Isolation of strong promoter by FACS screening

**Figure 3:**
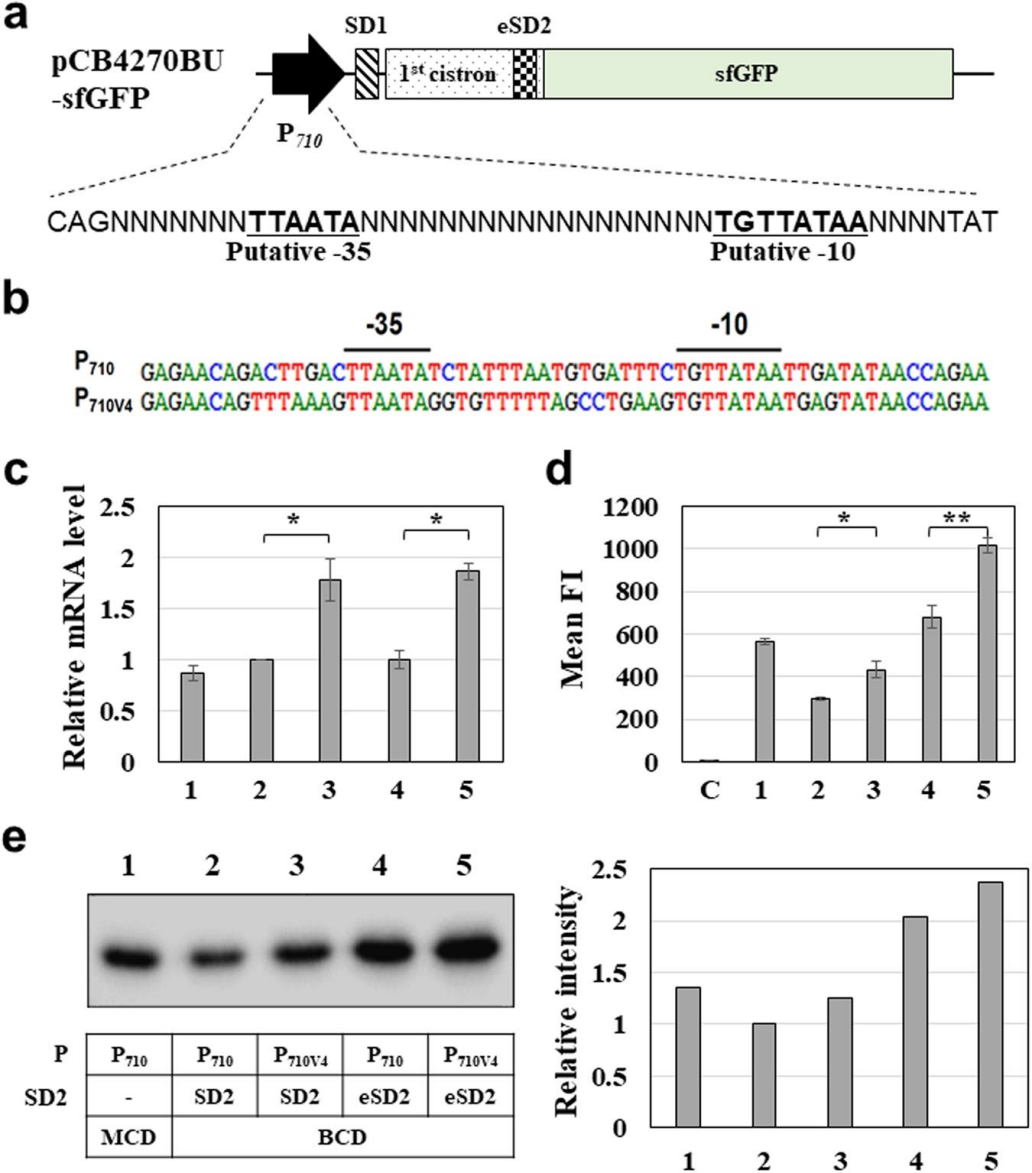
Isolation of strong P_710V4_ promoter. **(a)** Library construction for promoter engineering. Randomization regions of P_710_ are shown. Putative −35 and −10 regions of P_710_ are bold and underlined. **(b)** Sequence analysis of the isolated promoter. Putative −35 and −10 regions of the promoter are shown. **(c)** Evaluation of promoter strength by qRT-PCR. All error bars represent the value of standard deviation which were calculated from three repeated experiments. *p-value < 0.05 based on Student *t*-test. **(d)** FACS analysis of sfGFP fluorescence in the MCD and all BCDs. The bars represent the mean fluorescence intensity (Mean FI). C, pCB4270 (negative control. All error bars represent the value of standard deviation which were calculated from three repeated experiments. *p-value < 0.05, **p-value < 0.01 based on Student *t*-test **(e)** Western blotting of sfGFP expression in the MCD and all BCDs. Total fractions were loaded. A right panel shows the relative intensity of the bands in western blotting. Uncropped blot is shown in Supplementary Figure S11. (c), (d), and (e) 1, pCB4270-sfGFP; 2, pCB4270B-sfGFP; 3, pCB4270V4B-sfGFP; 4, pCB4270BU-sfGFP; 5, pCB4270V4BU-sfGFP.

A promoter is another important genetic element in gene expression; levels of gene expression highly depend on a promoter strength, making it necessary to find an optimal promoter for target gene expression^36^. In all the above constructs, P_710_ promoter isolated from *L*. *mesenteroides*^14^ was used, however its strength was not sufficient for the expression of sfGFP in *L*. *citreum*. Therefore, in addition to SD2 engineering, we modified P_710_ promoter to enable further increase of gene expression in the BCD. In pCB4270BU-sfGFP, promoter regions (29 bp) except for the putative −35 and −10 regions were randomized (Fig. 3a). In *E*. *coli*, a library of 2.6 × 10^7^ clones was obtained, and after transformation into *L*. *citreum*, we constructed a library of 3.4 × 10^5^ cells in *L*. *citreum*, which was used for FACS screening. In the first round, 2 × 10^7^ cells which were higher than the original library to prevent the loss of potential candidates, were screened and top 5% with high FI was selectively sorted (Supplementary Fig. S3). In the second round, top 1% of the total cells with high FI was selectively sorted and this procedure was repeated two more times. After the fourth round of sorting, 20 colonies were randomly selected and FI in each cell was analyzed by FACS. Among 20 clones, four candidates which showed higher FIs than other clones as well as the positive control harboring the original P_710_ promoter system (data not shown) were selected. Sequencing analysis revealed that the promoter sequences of all isolated clones were identical (Fig. 3b), and the isolated plasmid and promoter were named pCB4270V4BU-sfGFP and P_710V4_, respectively.

To determine the strength of the isolated promoter (P_710V4_), we constructed 4 different expression systems by combination of promoter (P_710_ and P_710V4_) and SD (SD2 and eSD2) parts, and the mRNA transcription level and protein level in each BCD construct (P_710_-SD2, P_710V4_-SD2, P_710_-eSD2, and P_710V4_-eSD2) as well as MCD were compared. In the transcription analysis by qRT-PCR, the use of P_710V4_ (pCB4270V4B-sfGFP and pCB4270V4BU-sfGFP) showed 1.7–1.9 fold higher transcription level than those of expression systems with original P_710_ (pCB4270B-sfGFP and pCB4270BU-sfGFP) regardless of SD sequence (Fig. 3c), which results clearly indicate the higher strength of the isolated promoter (P_710V4_) compared with the original promoter (P_710_). In addition, the sfGFP expression levels were investigated by FACS analysis and western blotting. In both analysis, expression levels in the BCD systems gradually increased by adding the synthetic parts, and the use of P_710V4_ exhibited higher expression levels than those of the original promoter (P_710_) (Fig. 3d,e, Supplementary Fig. S4). Among all examined systems, the combination of P_710V4_ and eSD2 exhibited the highest level of sfGFP expression which was 2.4-fold higher than that of the original BCD system (P_710_-SD2) (Fig. 3d,e). Also, we found that higher expression level could be obtained by employing eSD2 than SD2 (Fig. 3d,e) although the use of eSD2 did not increase mRNA transcript level (Fig. 3c). The higher gene expression level with eSD2 may be a result of the enhanced translational efficiency after transcription, which also indicate higher strength of eSD2 compared with SD2.

### Production of recombinant proteins using the engineered BCD with P_710V4_ and eSD2

**Figure 4:**
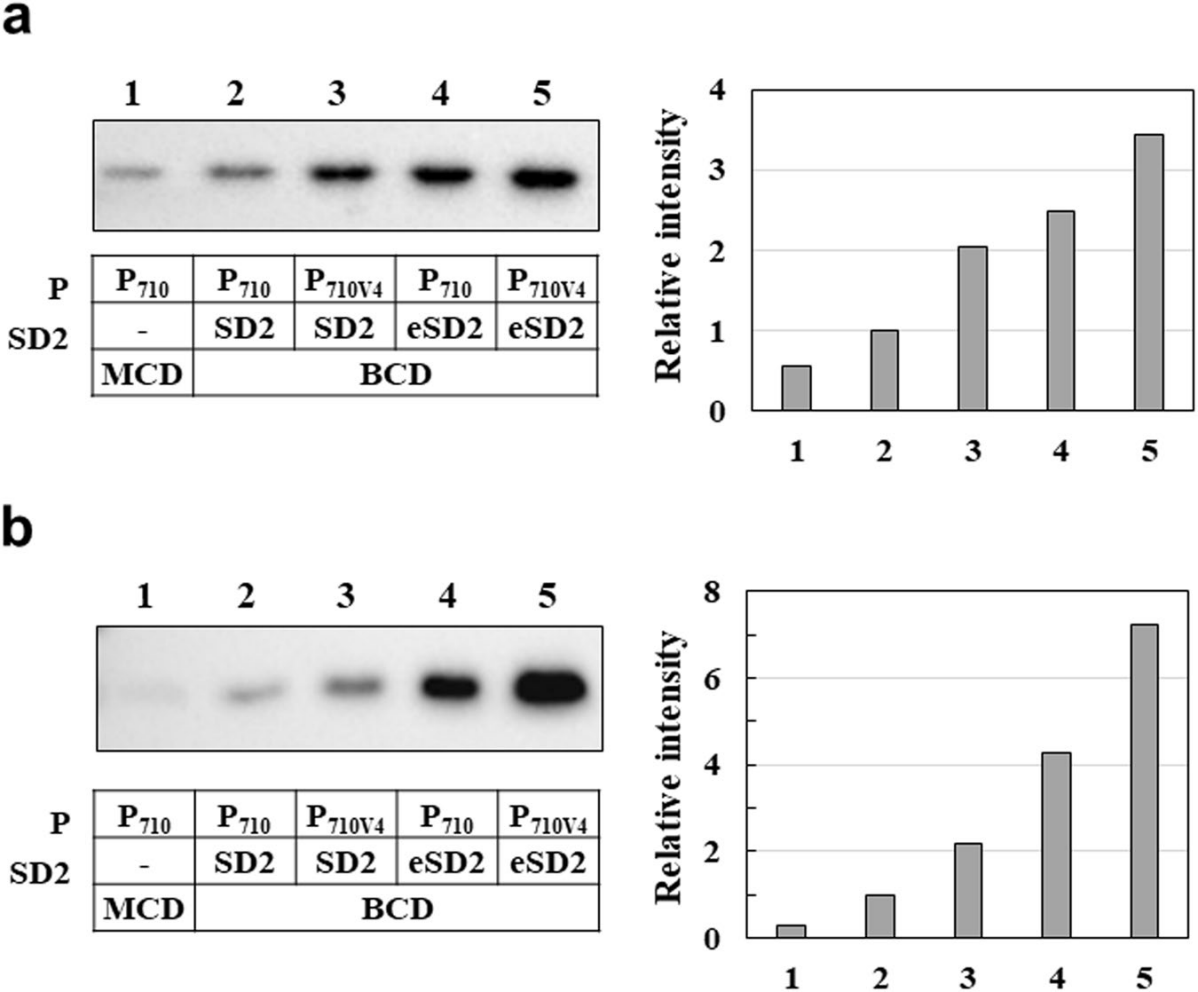
Comparison of expression levels of recombinant proteins in the MCD and all BCDs. **(a)** Western blot analysis of *L*. *citreum* producing GST. Total fractions were loaded. A right panel shows the relative intensity of the bands in western blotting. Lane 1, pCB4270-GST; lane 2, pCB4270B-GST; lane 3, pCB4270V4B-GST; lane 4, pCB4270BU-GST; lane 5, pCB4270V4BU-GST. **(b)** Western blot analysis of *L*. *citreum* producing hGH. Total fractions were loaded. A right panel shows the relative intensity of the bands in western blotting. Lane 1, pCB4270-hGH; lane 2, pCB4270B-hGH; lane 3, pCB4270V4B-hGH; lane 4, pCB4270BU-hGH; lane 5, pCB4270V4BU-hGH. (a) and (b), Uncropped images are shown in Supplementary Figure S11.

To explore usefulness of the engineered BCD with P_710V4_ and eSD2 in *L*. *citreum*, we examined the production of three recombinant proteins: (i) glutathione-S-transferase (GST), (ii) human growth hormone (hGH), and (iii) α-amylase. First, for the production of the relatively small (26 kDa) and soluble GST, four plasmids pCB4270B-GST, pCB4270V4B-GST, pCB4270BU-GST, and pCB4270V4BU-GST, which employed P_710_-SD2, P_710V4_-SD2, P_710_-eSD2, and P_710V4_-eSD2 in the BCD, respectively, were constructed. After cultivation in shake flask, the production yield of GST in each expression system was analyzed by SDS-PAGE followed by western blotting. As shown in Fig. 4a, gene expressions in all BCD systems were higher than that in the MCD system (pCB4270-GST). Also, we found the expression levels of GST were increased by combining each isolated part (P_710V4_ and eSD2) and original part (P_710_ and SD2) (Fig. 4a). Among four BCDs, the BCD system with P_710V4_-eSD2 (pCB4270V4BU-GST) exhibited 3.4-fold higher production of GST than that in the original BCD (Fig. 4a, Supplementary Fig. S5). In each BCD and MCD system, the transcription level was also determined by qRT-PCR and, similarly to sfGFP expression (Fig. 3c), higher transcriptions were observed under the P_710V4_ than the original P_710_ irrespective of SD sequence (Supplementary Fig. S9). Additionally, it was confirmed that the activity of soluble GST showed good correlation with expression strength (Supplementary Fig. S6). Among all examined systems, the combination of P_710V4_ and eSD2 also exhibited the highest activity, which was 4-fold and 18-fold higher than those of original BCD and MCD, respectively.

Next, we examined the expression of hGH which plays a pivotal role in human development and has been used to treat hypopituitarism and related diseases^37,38^. For the expression of hGH gene in the MCD and each BCD, five plasmids including pCB4270-hGH, pCB4270B-hGH, pCB4270V4B-hGH, pCB4270BU-hGH and pCB4270V4BU-hGH were constructed, respectively. In western blotting analysis, all BCDs exhibited higher levels of expression than that of the MCD in which a very faint band was detected (Fig. 4b, Supplementary Fig. S7). Similar to GST, it was clearly observed that the expression levels of hGH in the BCDs were increased as the engineered parts (P_710V4_ and eSD2) were added. Among all expression systems, the BCD system with P_710V4_-eSD2 exhibited 7.2-fold higher production of hGH than that in the original BCD. In the transcription level analysis by qRT-PCR, we also got similar results as sfGFP and GST expression: higher transcriptions under the P_710V4_ than the original P_710_ and the use of eSD2 did not effect on the transcription level (Supplementary Fig. S9).

**Figure 5:**
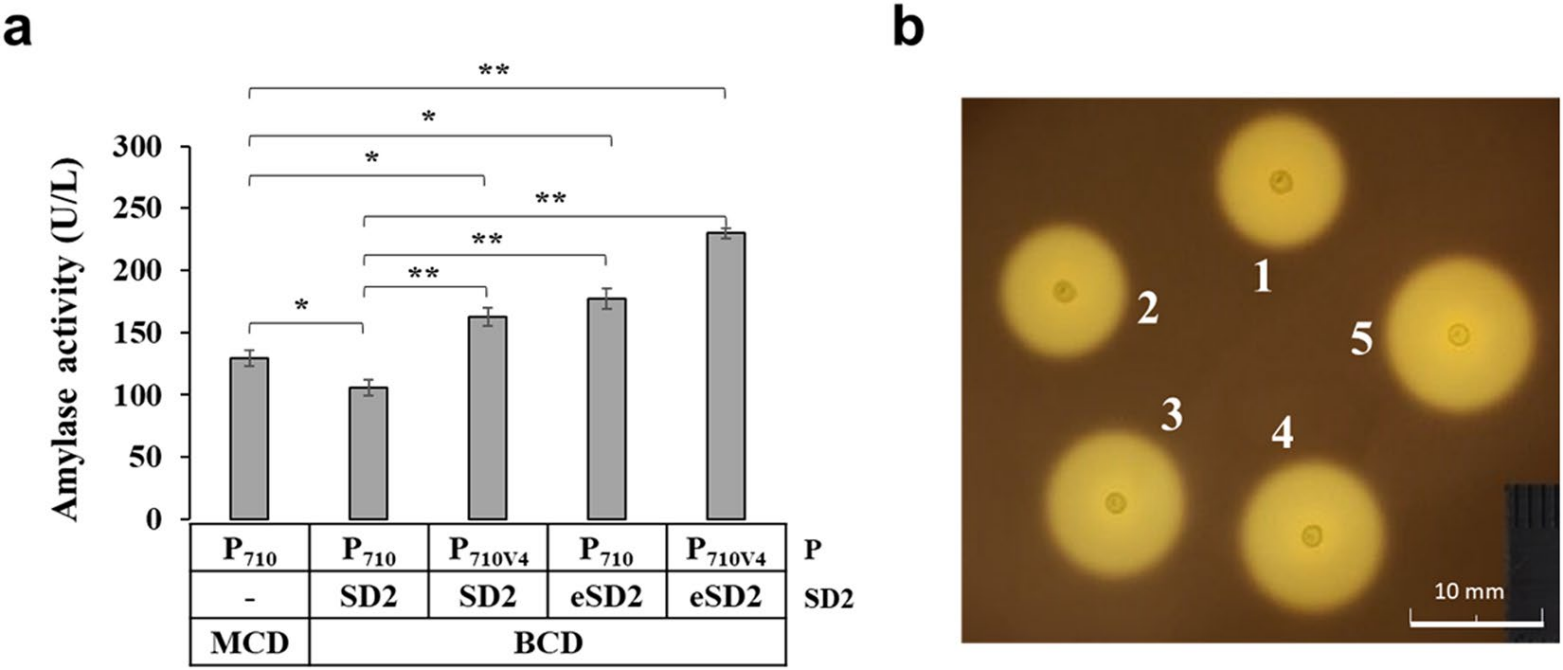
Comparison of α-amylase activity in the MCD and all BCDs. **(a)** Quantitative assay of α-amylase activity. The bars (from left to right) represent pCB4270-amy, pCB4270B-amy, pCB4270V4B-amy, pCB4270BU-amy, and pCB4270V4BU-amy, respectively. All experiments were done in triplicate. *p-value < 0.05, **p-value < 0.01 based on Student *t*-test. **(b)** Detection of α-amylase activity on 0.5% starch MRS-chloramphenicol plate. After incubation, the halos on the plate were visualized by staining with iodine solution. Spots 1 to 5, *L*. *citreum* harboring pCB4270-amy, pCB4270B-amy, pCB4270V4B-amy, pCB4270BU-amy, and pCB4270V4BU-amy, respectively. Uncropped photograph is shown in Supplementary Figure S12.

Finally, we examined the usability of the engineered BCD systems for the secretory production of recombinant protein in *L*. *citreum*. As a model protein, α-amylase (105 kDa) which can degrade starch to glucose was used. For the secretory production, signal peptide of α-amylase was used and the gene was cloned into four different BCDs, yielding pCB4270B-amy, pCB4270V4B-amy, pCB4270BU-amy, and pCB4270V4BU-amy. After cultivation in shake flask, the α-amylase activity in each system was determined with the culture supernatant sample. In the activity assay, cells harboring pCB4270V4BU-amy showed approximately 2.2-, 1.4-, and 1.3-fold higher activity (230 U/L) compared to cells harboring pCB4270B-amy (105.8 U/L), pCB4270V4B-amy (162.6 U/L) or pCB4270BU-amy (177.1 U/L), respectively (Fig. 5a). Interestingly, we found that the secretory production of α-amylase in the MCD was slightly higher (129.7 U/L) than that of the original BCD (pCB4270B-amy) but lower than that of other BCDs. The activity of α-amylase was also determined by the starch plate assay, in which the halo size indicates the activity of the secreted α-amylase. Similar to activity assay, the BCD with P_710V4_ and eSD2 exhibited the biggest halo (*d* = 10.9 ± 0.3 mm) than those of other BCDs and MCD. Also, the MCD exhibited a little bigger halo (*d* = 9.4 ± 0.3 mm) than that of the original BCD (*d* = 9.1 ± 0.2 mm), but much smaller than other BCDs (Fig. 5b, Supplementary Table S3). The production levels of α-amylase in all systems into culture medium, were also determined by SDS-PAGE analysis and, a pattern similar to that found in the activity assays of α-amylase was observed (Supplementary Fig. S8). Transcription level in each BCD system was analyzed by qRT-PCR, and similar to earlier models, the use of eSD2 did not give any positive effect on the transcription level, but the use of strong promoter (P_710V4_) allowed higher transcription of α-amylase gene (Supplementary Fig. S9). Collectively, we concluded that the engineered eSD2 and P_710V4_ in our BCD contributed to the higher and reliable production of recombinant proteins in *L*. *citreum*.

## Discussion

*Leuconostoc* sp. and many LABs have attracted attention as a host for the production of various recombinant proteins as well as for medical applications such as use as a live vehicle^9,39,40^. To further improve the potential of LABs, development of various genetic tools useful for engineering of gene expression was increasingly required. In this study, we developed a BCD system in which two genetic elements (promoter and SD) were successfully engineered for the enhanced production of recombinant proteins in *L*. *citreum*. Screening of the SD2 random library identified an engineered SD (eSD2) containing four mutations (5′-G**G**AA**G**GGAGG**GT**-3′) compared to the original SD2 sequence and clearly demonstrated that the use of eSD2 allowed higher production of recombinant proteins. However, no differences were noted in the mRNA transcript levels and stability upon comparing use of eSD2 and the original SD2 (Supplementary Fig. S2), indicating that eSD2 did not positively influence transcription. Another possible reason for the increase in protein production with eSD2 could be the enhanced interaction between 16S-rRNA and eSD2 in the ribosome, which increases the translation initiation rate and consequent protein production^40^. To confirm this possibility, it is necessary to check the binding affinity based on the hybridization energy of eSD2 sequence to 16S rRNA of the host^21,41^. However, since the anti-SD sequence of 16S rRNA of *L*. *citreum* has not been identified yet, the corresponding hybridization energy cannot be determined here. We also hypothesize that the sequence changes in eSD2 are more preferable for translation in *L*. *citreum*. It is known that AT-rich sequence is preferred in UTR^32,42^, although recently, it was also reported that less cytosine and more guanine sequences in the UTR increase translation in *E*. *coli*^43^. Compared with the original sequence (5′- GCAAAGGAGGTG-3′), the engineered eSD2 contains three more guanines and lacks cytosine, which might positively affect the translation rate.

In addition to eSD2, P_710_ promoter was also engineered by FACS screening strategy, and approx. 2-fold stronger promoter (P_710V4_) compared with original P_710_ promoter was isolated. From the transcription level analysis by qRT-PCR, the higher strength of P_710V4_ than the original P_710_ was clearly confirmed (Supplementary Fig. S9), and using the combination of the engineered P_710V4_ and eSD2, the enhanced gene expression in *L*. *citreum* was successfully demonstrated with three recombinant protein models (GST, hGH, and α-amylase). In all cases, the expression level in the BCD with P_710V4_-eSD2 was much higher than that of the original BCD as well as the MCD, indicating that the engineered BCD system can be a potential platform for the enhanced and reliable gene expression in *L*. *citreum*. To improve the potential of the present BCD system further, the engineering of the leader peptide in the 1^st^ cistron can be considered. Recently, Zhao *et al*.^24^ isolated a highly-expressed gene based on the proteome analysis, and used its N-terminal peptide as the 1^st^ cistron in BCD to successfully demonstrate the drastic increase of 2^nd^ cistron gene expression in BCD. In the present BCD system, we used a synthetic peptide, developed for the expression in *E*. *coli*, as a leader peptide. Although it was effective as shown here, we think the current BCD system can be further improved by employing high expressing leader peptide. In addition to our synthetic parts, we also consider the modification of DNA sequence of target gene for further increase of gene expression. The overall production yields of proteins are generally correlated with translation efficiency that is determined by translation initiation and elongation rates. Although we successfully demonstrated that our synthetic parts (eSD2) in the BCD system are highly effective on the translation initiation of target genes, those synthetic parts cannot control the translation elongation rate which is mainly dependent on the sequences of target gene (codon usage); the presence of rare codons or codon bias in the target gene, often cause the slow translation elongation^44,45^. By the modification of target gene sequence (i.e codon optimization), the translation elongation rate can be increased, and the overall production yield can be further increased by the combination with our synthetic parts.

**Figure 6:**
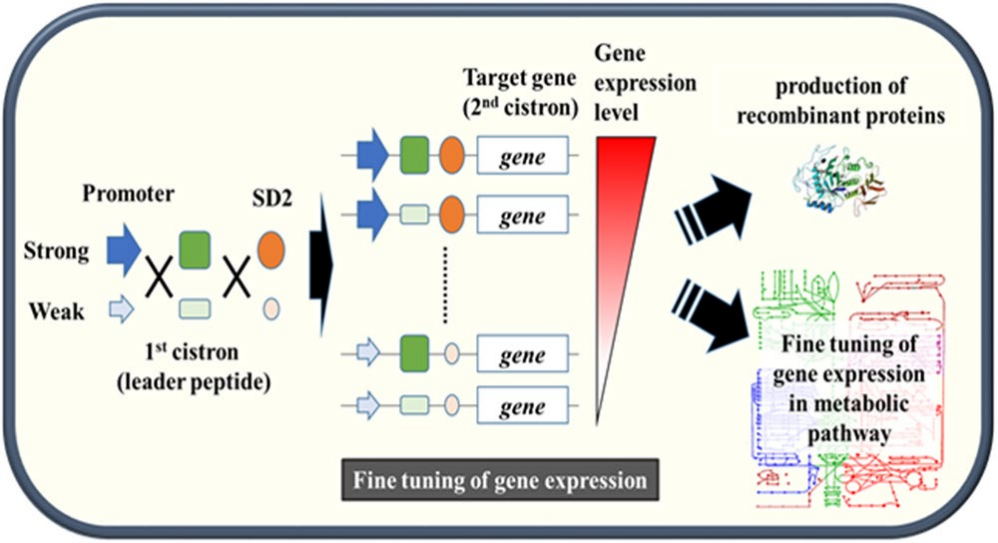
Fine tuning of the expression of key genes using various BCDs.

Compared with the conventional MCD system, the BCD system has several beneficial features. First, using the well-expressed leader peptide (1^st^ cistron), the expression of the target gene (2^nd^ cistron) can be maintained at a high level, irrespective of the target gene sequences, which allows reliable gene expression in *L*. *citreum*. When the MCD system was used, the three examined genes exhibited varying levels of gene expression. Expression of GST and hGH in the MCD was very weakly detected by western blotting (Fig. 4), but the expression of α-amylase and sfGFP was higher than that in the original BCD (Figs 3 and 5). In contrast, all examined genes in the BCD systems were similarly expressed at high levels (Figs 4 and 5). Second, using the BCD, the expression of each target gene can be controlled more precisely, which is useful for the development of a tunable gene expression system in the host. As shown here (Fig. 4), different levels of gene expression for the target gene (2^nd^ cistron) can be easily achieved by employing different genetic components (promoter and SD). For example, strong expression can be achieved by the assembly of the engineered parts (P_710V4_ and eSD2), and moderate or lower gene expression can be achieved by the combination of the original and engineered parts (P_710_-eSD2 or P_710V4_-SD2) or all original parts (P_710_ and SD2) which have relatively weak strength (Fig. 6). Particularly, the engineered BCD system can be a potential tool in the production of insoluble proteins. When we checked the solubility of proteins in all BCD systems, all examined proteins (sfGFP, GST, hGH and amylase) showed high solubilities, and the highest production of soluble proteins could be achieved with strongest parts (P_710V4_-eSD2) (Supplementary Figs S4, S5, and S7). However, if the protein is relatively insoluble, the strong expression system may lead to the formation of inclusion bodies which are not functional, and it is necessary to optimize the expression level for the production of soluble proteins. Using our BCD systems, the expression level can be tuned, and the proper expression system can be developed for the production of soluble proteins. Currently, systems and synthetic biology approaches for the engineering of bacterial hosts highly require various genetic parts capable of controlled/tunable expression^46–48^. Using these controllable genetic parts, new or existing biosynthetic pathways in the hosts can be optimized through fine tuning of the expression of key genes in biosynthetic pathways. We also propose that our engineered genetic parts in the BCD can be a useful tool for synthetic biology-based engineering of *L*. *citreum*. In addition to promoter and SD2, 1^st^ cistron can be designed further, and by combination of those genetic parts, fine tuning of the expression of key genes in metabolic pathway can be achieved in *L*. *citreum* (Fig. 6).

In conclusion, we successfully constructed a BCD system suitable for high production of recombinant proteins in *L*. *citreum*. For improved and reliable expression, the promoter and SD2 were engineered by FACS-based high-throughput screening strategy, and the usefulness of the engineered BCD system for enhanced and reliable gene expression in *L*. *citreum* was also successfully demonstrated with three protein models including GST, hGH, and α-amylase. Although BCD system with translational coupling was already reported in a few LAB strains, there has been no attempt to develop synthetic parts (promoter and SD sequence) for BCD system in LABs yet^27,28^. To the best of our knowledge, this is the first report on the engineering of BCD with synthetic parts, which are useful for reliable and tunable expression in *L*. *citreum* as well as other LABs. We believe that our engineered BCD system can be a potential genetic tool for synthetic biology-based engineering of *L*. *citreum*. In addition, screening strategies as described here can be used to isolate more potent or regulatable BCDs which will contribute to the engineering of LAB.

## Methods

### Bacterial strains and culture conditions

*E*. *coli* XL1-Blue was used as a host for the cloning, maintenance of plasmids, and library construction. *L*. *citreum* CB2567 was used for library screening and protein production. *E*. *coli* was cultivated in Lysogeny broth (LB) medium (BD, Franklin Lakes, NJ, USA) at 37 °C with shaking (200 rpm). *L*. *citreum* was cultivated in Lactobacilli MRS broth (BD) at 30 °C with shaking (200 rpm). Ampicillin (100 mg/L) and chloramphenicol (10 mg/L) were added in the cultivation of *E*. *coli* and *L*. *citreum*, respectively.

### Plasmids construction

The plasmids and primers used in this study are listed in Supplementary Tables S1 and S2. Polymerase chain reaction (PCR) was carried out using C1000TM Thermal Cycler (Bio-Rad, Richmond, CA, USA) and PrimeSTAR HS Polymerase (TAKARA BIO Inc., Shiga, Japan). The sfGFP gene was amplified from pCB4270-sfGFP by PCR with four primers (F1-BCD-sfG, F2-BCD-sfG, F3-BCD-sfG, and R-sfG). The PCR product was digested by two restriction enzymes (*Hin*dIII and *Sal*I) and cloned into pCB4270-sfGFP, yielding pCB4270B-sfGFP. To verify the eSD2 activity, sfGFP gene was amplified by PCR with three primers (F1-mSD2-sfG, F2-BCD-et, and R-sfG). pCB4270B-sfGFP was digested by two restriction enzymes (*Xho*I and *Not*I) and the small fragment was cloned into pCB4270V4BU-sfGFP, yielding pCB4270V4B-sfGFP. The PCR product was digested with two restriction enzymes (*Xho*I and *Sal*I) and cloned into pCB4270B-sfGFP, generating pCB4270BM-sfGFP. For expression, GST gene was amplified from pCB4270-GST by PCR with two set of primers: F1-BCD-GST, F2-BCD-et, and R-GST or F1-mSD2-GST, F2-BCD-et, and R-GST. The former PCR product was digested with *Xho*I and *Not*I and cloned into pCB4270B-sfGFP and pCB4270V4B-sfGFP to yield pCB4270B-GST and pCB4270V4B-GST. The latter was digested with same restriction enzymes and cloned into pCB4270BU-sfGFP and pCB4270V4BU-sfGFP generating pCB4270BU-GST and pCB4270V4BU-GST, respectively. For the expression of hGH gene in the MCD, the gene^38^ was chemically synthesized (General Biosystems Inc., NC, USA), and amplified by PCR with three primers (F1-P710-hGH, F2-P710, and R-hGH). The PCR product was digested with *Hin*dIII and *Not*I and ligated with pCB4270-sfGFP, resulting in pCB4270-hGH. For the expression in the BCD, hGH gene was amplified from pCB4270-hGH by PCR with two sets of primers: F1-BCD-hGH, F2-BCD-et, and R-hGH or F1-mSD2-hGH, F2-BCD-et, and R-hGH. The former PCR product was digested with *Xho*I and *Not*I and cloned into pCB4270B-sfGFP and pCB4270V4B-sfGFP to yield pCB4270B-hGH and pCB4270V4B-hGH. The latter was digested with the same restriction enzymes and cloned into pCB4270BU-sfGFP and pCB4270V4BU-sfGFP to yield pCB4270BU-hGH and pCB4270V4BU-hGH, respectively. For the expression of α-amylase from *L*. *amylovorus*, the gene was amplified from pCB4270-amy by PCR with one set of primers (F1-BCD-amy, F2-BCD-et, and R-amy) or using another set of primers (F1-mSD2-amy, F2-BCD-et, and R-amy). The former PCR product was digested with *Xho*I and *Not*I and cloned into pCB4270B-sfGFP and pCB4270V4B-sfGFP to yield pCB4270B-amy and pCB4270V4B-amy. The latter PCR product was digested with same restriction enzymes and cloned into pCB4270BU-sfGFP and pCB4270V4BU-sfGFP generating pCB4270BU-amy and pCB4270V4BU-amy, respectively.

### Construction of SD2 and P_710_ promoter libraries

For the construction of SD2 library, pCB4270B-sfGFP was used as the template DNA, and SD2 region was randomized by PCR with three primers (F2-BCD-et, F1-BL, and R-sfG). The PCR product was digested with *Xho*I and *Sal*I and ligated into pCB4270B-sfGFP. For the construction of promoter library, pCB4270BU-sfGFP was used as the template, and P_710_ promoter region was randomized by PCR with two primers (F-P710RL and R-sfG). PCR product was digested with *Hin*dIII and *Sal*I, and further was ligated into pCB4270BU-sfGFP. Each library was transformed into *E*. *coli* XL1-Blue by electroporation. The plasmids were purified from *E*. *coli* library using GeneAll® Hybrid-QTM Plasmid Rapidprep kit (GeneAll, Seoul, Korea) and transformed into *L*. *citreum* CB2567 by electroporation using the following parameters: 25 μF, 1.0 kV, and 400 Ω^49^. Transformed cells were recovered by incubation on MRS agar plates with 10 mg/L chloramphenicol at 30 °C for 36 h.

### Library screening by FACS

SD2 and promoter libraries were cultured overnight at 30 °C in MRS media. Further, cells were transferred to fresh MRS media at 1:50 dilution and grown at 30 °C with shaking (200 rpm). After cultivation for 10 h, cells were harvested by centrifugation (13,000 rpm, 5 min, 4 °C) and washed twice with phosphate-buffered saline (PBS, 135 mM NaCl, 2.7 mM KCl, 4.3 mM Na_2_HPO_4_, pH 7.2). After resuspension in the same buffer, high-fluorescent cells were identified using a high-speed flow cytometer (MoFlo XDP, Beckman Coulter, Miami, FL, USA). In FACS sorting, cells were selected on the basis of high fluorescence intensity detected through a 530/40 band-pass filter for the GFP emission spectrum. Sorted cells were immediately cultured in MRS media with chloramphenicol (10 mg/L) at 30 °C. After cultivation for 24 h, cells were reused for next-round of FACS sorting. The sorting was repeated until the highly fluorescent population was fully enriched.

### Protein preparation and analysis

After culturing in shake-flask at 30 °C for 10 h, cells were harvested by centrifugation (13,000 rpm for 5 min at 4 °C) and washed with PBS. Next, cells were disrupted by sonication (7 min with 5-s pulse and 3-s cooling time, 20% amplitude). After harvesting total fractions, insoluble pellets were removed by centrifugation (13,000 rpm for 5 min at 4 °C) to harvest soluble fractions. Protein samples were analyzed by 12% sodium dodecyl sulfate-polyacrylamide gel electrophoresis (SDS-PAGE) and western blotting. For western blotting, the proteins on the SDS-PAGE gel were transferred to a polyvinyl difluoride (PVDF) membrane (Roche, Basel, Switzerland). The membrane was blocked with 5% (w/v) skim-milk in Tris buffered saline with Tween-20 solution (TBS-T; 24.7 mM Tris, 137 mM NaCl, 2.7 mM KCl and 0.05% Tween-20) at room temperature for 1 h. The membrane was incubated with horseradish peroxidase (HRP)-conjugated goat anti-GFP antibody (Abcam, Cambridge, UK), HRP-conjugated anti-GST antibody (GE Healthcare, Uppsala, Sweden), or HRP-conjugated anti-His-antibody (Sigma-Aldrich, St. Louis, MO, USA) that was 1:4000 diluted with TBS-T buffer containing 5% skim-milk. After incubation at room temperature for 1 h, each membrane was washed 4 times with TBS-T. For immunodetection of the target proteins, an enhanced chemiluminescence reagent (ECL Prime, GE Healthcare) was used and finally the target protein bands were visualized on X-ray films.

### Quantitative reverse transcription PCR (qRT-PCR)

For the quantification of mRNA transcripts, reverse transcription followed by quantitative polymerase chain reaction (qRT-PCR) was performed. *L*. *citreum* harboring each plasmid was grown in MRS medium for 10 h, harvested by centrifugation (13,000 rpm for 5 min at 4 °C), and washed with PBS. For mRNA decay analysis, after 10 h cultivation, transcription was stopped by the addition of rifampicin (Sigma-Aldrich) to a final concentration of 500 μg/mL. The cells were harvested immediately before and after rifampicin addition. Total RNA was extracted from the cells using the Qiagen RNeasy Mini Kit (Qiagen, Valencia, CA, USA), and the purified RNA was stored at −80 °C until further use. The primer sets for performing qRT-PCR were designed using Primer3web (http://primer3.ut.ee), and the resulting primer sequences were listed in Table S2. One-step qRT-PCR was carried out by using One Step SYBR® PrimeScriptTM RT-PCR Kit (Takara Bio Inc.) according to the manufacture’s protocol. Thermal cycling and real-time monitoring of DNA synthesis were carried out using a Bio-Rad CFX Connect (Bio-Rad). The levels of mRNA was calculated by the comparative C_T_ method^50^. The transcript level of housekeeping gene (lactose dehydrogenase gene, *ldh*) was compared as an internal standard. The data that all the mRNAs were free of DNA contamination was shown in Supplementary Figure S10.

### GST activity assay

After culturing in shake-flask at 30 °C for 10 h, cells harboring each plasmid were harvested by centrifugation (13,000 rpm for 5 min at 4 °C) and washed with PBS. Next, cells were disrupted by sonication (7 min with 5-s pulse and 3-s cooling time, 20% amplitude). After removing the insoluble pellet by centrifugation, the soluble lysates were used for measuring the GST activity. The quantitative activity of GST was measured using Glutathione S-transferase (GST) Assay Kit (Sigma-Aldrich) following the manufacturer’s instructions.

### α-amylase activity assay

The activity of α-amylase secreted into the extracellular media was determined by the reaction with iodine solution on the starch plate^51^. The optical density of overnight cultures of *L*. *citreum* were measured and normalized, and the cultures were spotted onto MRS plates with 0.5% soluble starch and 10 mg/L chloramphenicol. After incubation at 30 °C for 24 h, 5 mL of Lugol’s iodine was evenly poured onto the plate. Clear halo zones around the colonies indicated starch degradation by the secreted α-amylase. The quantitative activity of amylase was measured using the EnzChek® Ultra Amylase Assay Kit (Thermo Fisher Scientific, Waltham, MA, USA) following the manufacturer’s instructions. Culture supernatants from 15 h cultivation were prepared, and the fluorescence intensity was detected with Infinite® F200 PRO (TECAN, Männedorf, Switzerland)^52^. A standard curve was constructed with α-amylase from *Bacillus* sp. (Sigma-Aldrich) according to the manufacturer’s instructions.

## Electronic supplementary material

Supplementary Information File #1
