# Supplementary material for: Development of bicistronic expression system for the enhanced and reliable production of recombinant proteins in Leuconostoc citreum
Source: Sci Rep. 2018 Jun 11;8:8852. doi: 10.1038/s41598-018-27091-z (PMC5995908; doi:10.1038/s41598-018-27091-z)
Supplement: Supplementary file 1 — Supplementary Information File #1 [file 41598_2018_27091_MOESM1_ESM.docx]

**Supplementary Information**

**Development of bicistronic expression system for the enhanced and reliable production of recombinant proteins in *Leuconostoc citreum***

Seung Hoon Jang^1^, Ji Won Cha^1^, Nam Soo Han^2^ & Ki Jun Jeong,^1,3^

^1^Department of Chemical and Biomolecular Engineering (BK21 Program), KAIST, 291 Daehak-ro, Yuseong-gu, Daejeon 34141, Republic of Korea;
telephone: +82-42-350-3934; fax: +82-42-350-3910; e-mail: kjjeong@kaist.ac.kr

^2^Brain Korea 21 Center for Bio-Resource Development, Division of Animal, Horticultural and Food Sciences, Chungbuk National University, Cheongju 28644, Republic of Korea

^3^KAIST Institute for the BioCentury, 291 Daehak-ro, Yuseong-gu, Daejeon 34141, Republic of Korea.

**Table S1.** Plasmids used in this study

| **Plasmid** | **Description** | **Reference** |
| --- | --- | --- |
| pCB4270 | High copy number plasmid, P_710_ | (Son et al., 2016) |
| pCB4270-sfGFP | Monocistronic, P_710_, sfGFP | (Son et al., 2016) |
| pCB4270B-sfGFP | Bicistronic, P_710_, sfGFP | This study |
| pCB4270V4B-sfGFP | Bicistronic, P_710V4_, sfGFP | This study |
| pCB4270BU-sfGFP | Bicistronic, P_710_, eSD2, sfGFP | This study |
| pCB4270BM-sfGFP | Bicistronic, P_710_, mutant SD2, sfGFP | This study |
| pCB4270V4BU-sfGFP | Bicistronic, P_710V4_, eSD2, sfGFP | This study |
| pCB4270-GST | Monocistronic, P_710_, GST | (Son et al., 2016) |
| pCB4270B-GST | Bicistronic, P_710_, GST | This study |
| pCB4270V4B-GST | Bicistronic, P_710V4_, GST | This study |
| pCB4270BU-GST | Bicistronic, P_710_, eSD2, GST | This study |
| pCB4270V4BU-GST | Bicistronic, P_710V4_, eSD2, GST | This study |
| pCB4270-hGH | Monocistronic, P_710_, hGH, His tag | This study |
| pCB4270B-hGH | Bicistronic, P_710_, hGH, His tag | This study |
| pCB4270V4B-hGH | Bicistronic, P_710V4_, hGH, His tag | This study |
| pCB4270BU-hGH | Bicistronic, P_710_, eSD2, hGH, His tag | This study |
| pCB4270V4BU-hGH | Bicistronic, P_710V4_, eSD2, hGH, His-tag | This study |
| pCB4270-amy | Monocistronic, P_710_, α-amylase | (Son et al., 2016) |
| pCB4270B-amy | Bicistronic, P_710_, α-amylase | This study |
| pCB4270V4B-amy | Bicistronic, P_710V4_, α-amylase | This study |
| pCB4270BU-amy | Bicistronic, P_710_, eSD2, α-amylase | This study |
| pCB4270V4BU-amy | Bicistronic, P_710V4_, eSD2, α-amylase | This study |

**Table S2.** Oligonucleotide primers used in this study

| **Primer** | **Sequence (5’ to 3’)** |
| --- | --- |
| F1-BCD-sfG | ACACTCGAGATGAAAGCAATTTTCGTACTGAAACATCTTAATCATGCAAAGGAGGTGTTTTAATGAGCAAAGGAGAAGAAC |
| F2-BCD-sfG | TAATTGATATAACCAGAAGGGCCCAAGTTCACTTAAAAAGGAGATCAACACTCGAGATGAAAGCAAT |
| F3-BCD-sfG | CGAAGCTTGAGAACAGACTTGACTTAATATCTATTTAATGTGATTTCTGTTATAATTGATATAACCAGAAGGG |
| R-sfG | GTGTCGACAGGTAATGGTTG |
| F1-BL-sfG | GTACTGAAACATCTTAATCATNNNNNGGAGGNNTTTTAATGAGCAAAGGAGAAGAAC |
| F1-mSD2-sfG | GTACTGAAACATCTTAATCATGGAAGGGAGGGTTTTTAATGAGCAAAGGAGAAGAAC |
| F2-BCD-et | ATCTCGAGATGAAAGCAATTTTCGTACTGAAACATCTTAATCAT |
| F-P710RL | CCAAGCTTGAGAACAGNNNNNNNTTAATANNNNNNNNNNNNNNNNNNTGTTATAANNNNTATAACCAGAAGGGCCCAA |
| F1-BCD-GST | GTACTGAAACATCTTAATCATGCAAAGGAGGTGTTTTAATGTCCCCTATACTAGGTTA |
| F1-mSD2-GST | GTACTGAAACATCTTAATCATGGAAGGGAGGGTTTTTAATGTCCCCTATACTAGGTTA |
| R-GST | GGGCGGCCGCTTATTAATCCGATTTTGGAGGATGG |
| F1-BCD-hGH | GTACTGAAACATCTTAATCATGCAAAGGAGGTGTTTTAATGTTCCCGACGATCCCT |
| F1-mSD2-hGH | GTACTGAAACATCTTAATCATGGAAGGGAGGGTTTTTAATGTTCCCGACGATCCCT |
| F2-P710 | GAAAGCTTGAGAACAGACTTGACTTAATATCTATTTAATGTGATGTCTGTTATAATTGATATAACCAGA |
| F1-P710-hGH | GTGATGTCTGTTATAATTGATATAACCAGAAGAAAGGATAGAAAAAATGTTCCCGACGATCCCT |
| R-hGH | CTGCGGCCGCTTATTAGTGGTGGTGGTGGTGGTGGAAGCCGCAGCTGCCCT |
| F1-BCD-amy | GTACTGAAACATCTTAATCATGCAAAGGAGGTGTTTTAATGAAAAAAAAGAAAAGTTTCTGG |
| F1-mSD2-amy | GTACTGAAACATCTTAATCATGGAAGGGAGGGTTTTTAATGAAAAAAAAGAAAAGTTTCTGG |
| R-amy | TGGCGGCCGCTTATTACGAATTGCTTGATGTGCTG |
| F-RT-ldh | GGTCAGTTAATGGGCTTGAT |
| R-RT-ldh | CAGCAATACTTGGTGTCTCA |
| F-RT-sfGFP | TGCTACAAACGGAAAACTCA |
| R-RT-sfGFP | GCGTTCCTGTACATAACCTT |
| F-RT-GST | GCGCGATGAAGGTGATAAAT |
| R-RT-GST | ATTGAAATCTCTGCACGCTC |
| F-RT-hGH | AAAACCCACAAACGTCACTC |
| R-RT-hGH | CGCCATATACCAACGAGTTC |
| F-RT-amy | AAAGTTGGAAATAGCGGGTC |
| R-RT-amy | TCATTCAGAGTTGCATCGAC |

**Table S3.** Diameter and area of halos on the starch-agar plate. All data represent the mean value with standard deviation from three repeated experiments.

| No | plasmids | Diameter (mm) | Area (mm^2^) |
| --- | --- | --- | --- |
| 1 | pCB4270-amy | 9.4 ± 0.3 | 69.9 ± 4.2 |
| 2 | pCB4270B-amy | 9.1 ± 0.2 | 65.5 ± 3.3 |
| 3 | pCB4270V4B-amy | 9.9 ± 0.3 | 77.0 ± 4.7 |
| 4 | pCB4270BU-amy | 10.3 ± 0.3 | 82.8 ± 4.9 |
| 5 | pCB4270V4BU-amy | 10.9 ± 0.3 | 93.9 ± 5.2 |


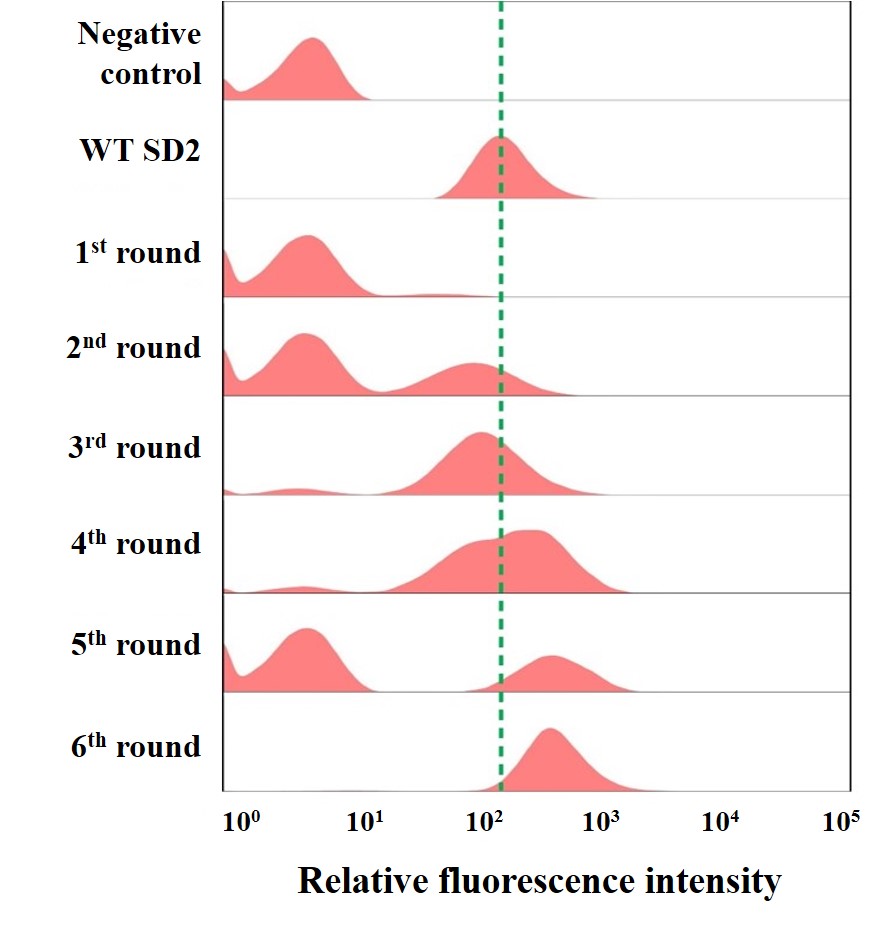


**Supplementary Figure S1.** FACS screening of the SD2 library. pCB4270 and pCB4270B-sfGFP were used as a negative control and original SD2 (WT-SD2), respectively. Green dash line indicates the peak in the histogram of the BCD with original SD2 (WT-SD2).


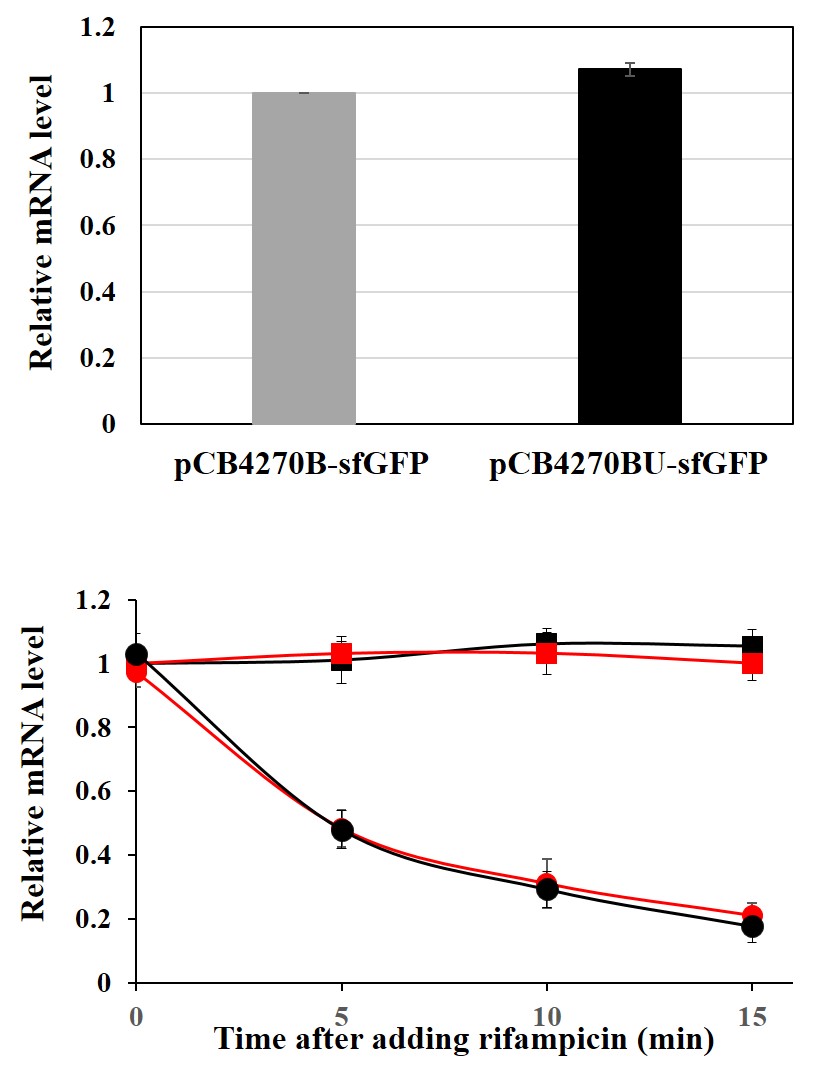


**A**

**B**

**Supplementary Figure S2.** (A) mRNA levels of pCB4270B-sfGFP and pCB4270BU-sfGFP. (B) Evaluation of mRNA decay of pCB4270B-sfGFP and pCB4270BU-sfGFP by qRT-PCR. Symbols: Circles and squares represent the rifampicin treated and untreated samples, respectively. Red and black symbols represent pCB4270B-sfGFP and pCB4270BU-sfGFP, respectively. All error bars represent the value of standard deviation which was calculated from three times repeated experiments.

**
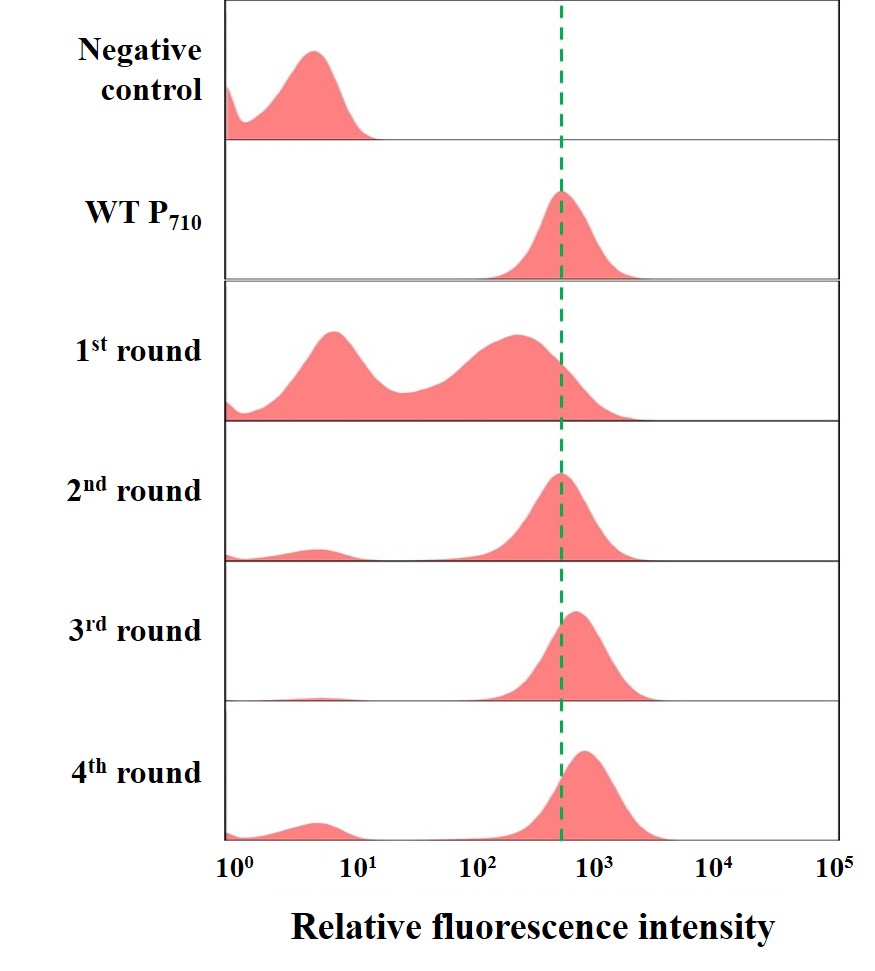
**

**Supplementary Figure S3.** FACS screening of the P_710_ library. pCB4270 and pCB4270BU-sfGFP were used as a negative control and wild-type P_710_ (WT-P_710_), respectively. Green dash line indicates the peak in the histogram of the BCD with original P_710_ (WT-P_710_).


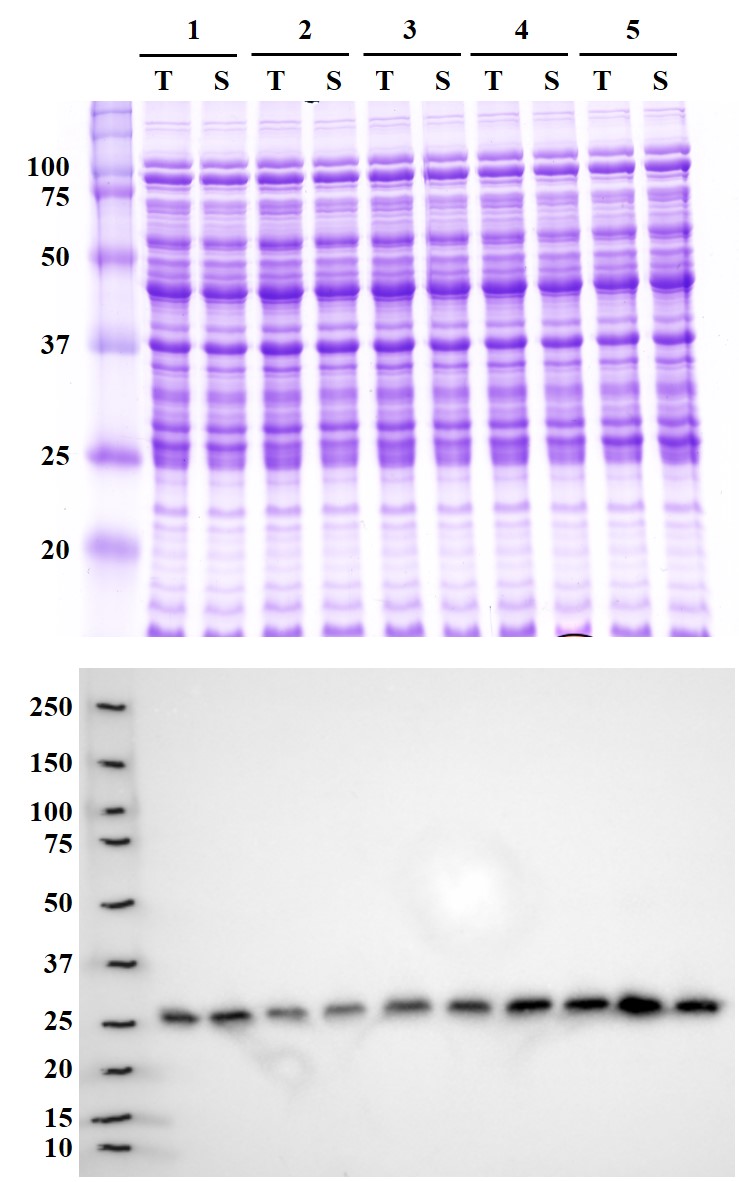


**Supplementary Figure S4.** Investigation of sfGFP solubilities in the MCD and all BCDs. The panels show a coomassie blue stained SDS-PAGE gel (top panel) and a western blot (bottom panel). Lanes 1 to 5, pCB4270-sfGFP, pCB4270B-sfGFP, pCB4270V4B-sfGFP, pCB4270BU-sfGFP, and pCB4270V4BU-sfGFP; lanes T and S represent total and soluble protein fractions, respectively.

**
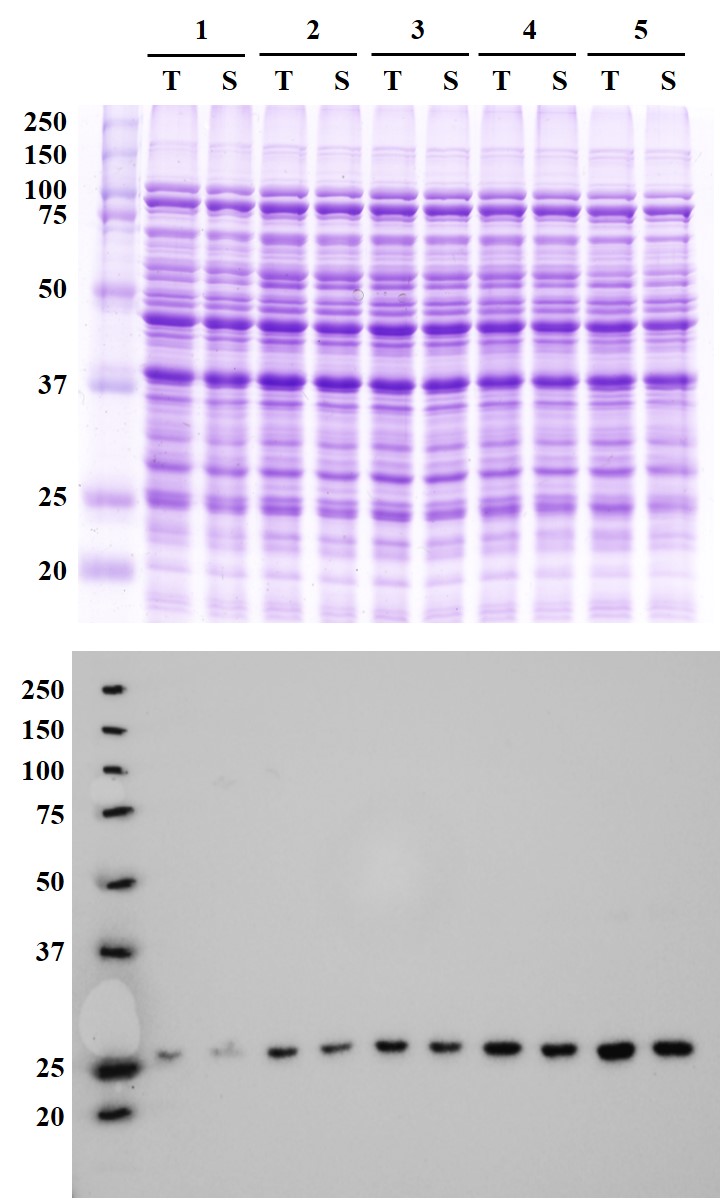
**

**Supplementary Figure S5.** Investigation of GST solubilities in the MCD and all BCDs. The panels show a coomassie blue stained SDS-PAGE gel (top panel) and a western blot (bottom panel). Lanes 1 to 5, pCB4270-GST, pCB4270B-GST, pCB4270V4B-GST, pCB4270BU-GST, and pCB4270V4BU-GST; lanes T and S represent total and soluble protein fractions, respectively.


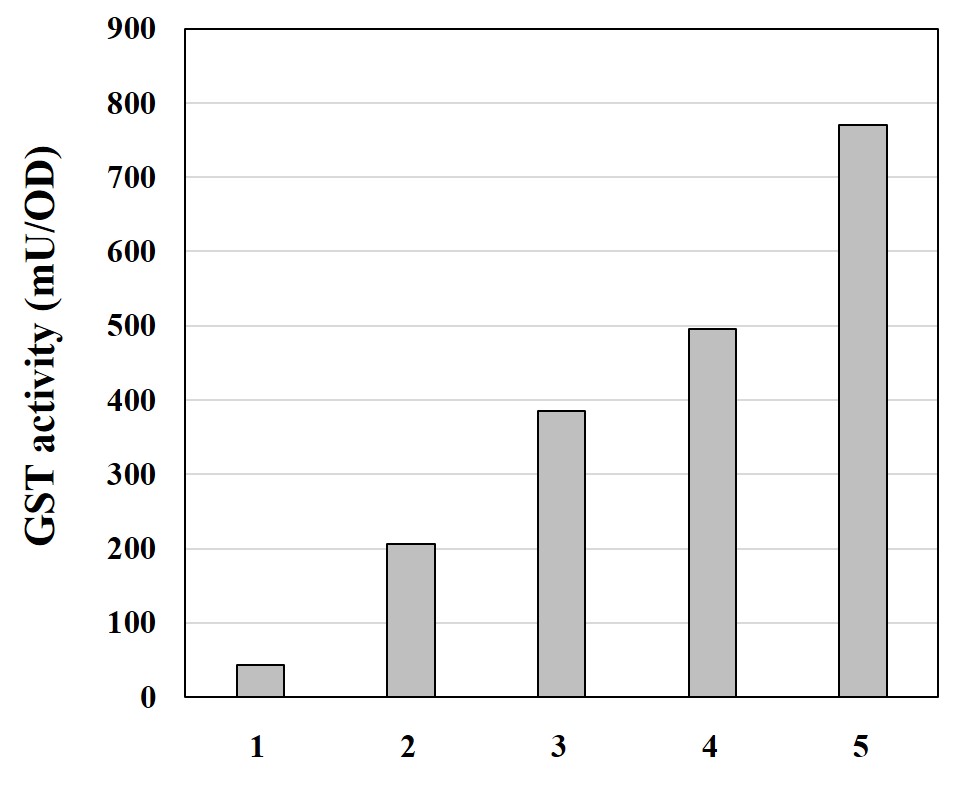


**Supplementary Figure S6.** GST activity in the MCD and all BCDs. The soluble lysates were used for measurement of GST activity. #1, pCB4270-GST; #2, pCB4270B-GST; #3, pCB4270V4B-GST; #4, pCB4270BU-GST; #5, pCB4270V4BU-GST.


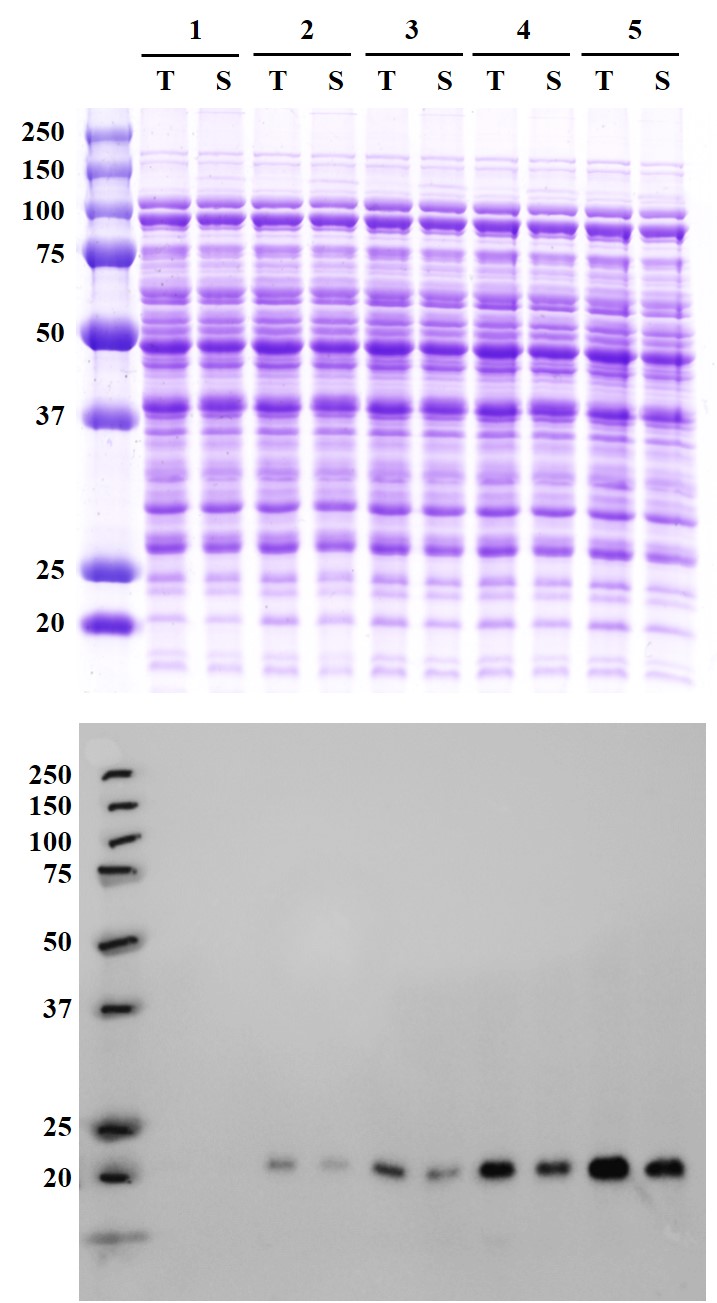


**Supplementary Figure S7.** Investigation of hGH solubilities in the MCD and all BCDs. The panels show a coomassie blue stained SDS-PAGE gel (top panel) and a western blot (bottom panel). Lanes 1 to 5, pCB4270-hGH, pCB4270B-hGH, pCB4270V4B-hGH, pCB4270BU-hGH, and pCB4270V4BU-hGH; lanes T and S represent total and soluble protein fractions, respectively.


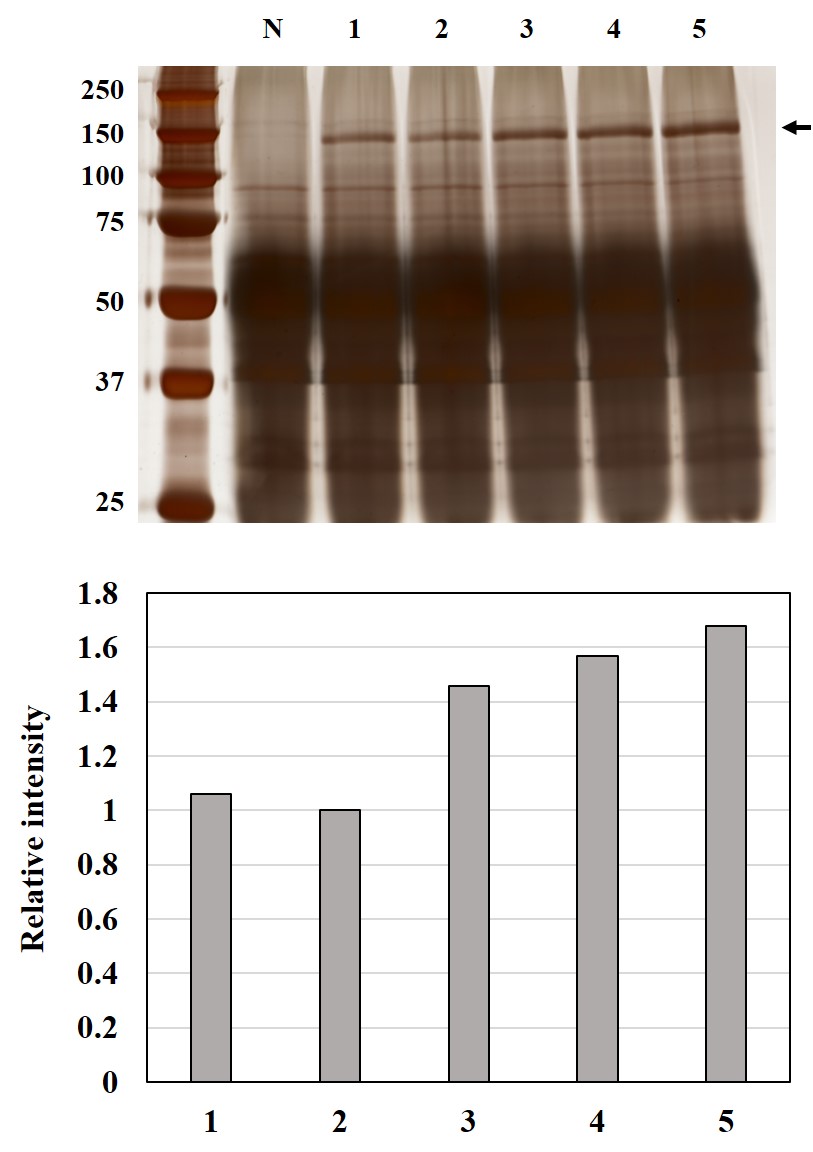


**Supplementary Figure S8.** Investigation of expression levels of α-amylase in the MCD and all BCDs. The panels show silver stain of SDS-PAGE gel (top panel) and the relative intensity of the bands in SDS-PAGE gel. Lane N, negative control (pCB4270); lanes 1 to 5, pCB4270-α-amylase, pCB4270B-α-amylase, pCB4270V4B-α-amylase, pCB4270BU-α-amylase, and pCB4270V4BU-α-amylase, respectively. An arrow indicates α-amylase. After culturing in shake-flask at 30 °C for 24 h, the culture supernatant was obtained by centrifugation (13,000 rpm for 5min at 4 °C) and vigorously mixed with two volumes of cold acetone. After incubation at −20 °C for 12 h, the protein samples were then precipitated by centrifugation at 13,000 rpm for 30 min at 4 °C. After resuspension of the pellets, protein samples were analyzed by 8% SDS-PAGE. After gel electrophoresis, extracellular proteins were visualized using a silver staining kit (GE Healthcare) and the stained gels were scanned using a UMAX PowerLook 2100XL Scanner (UMAX Technologies, Taipei, Taiwan).


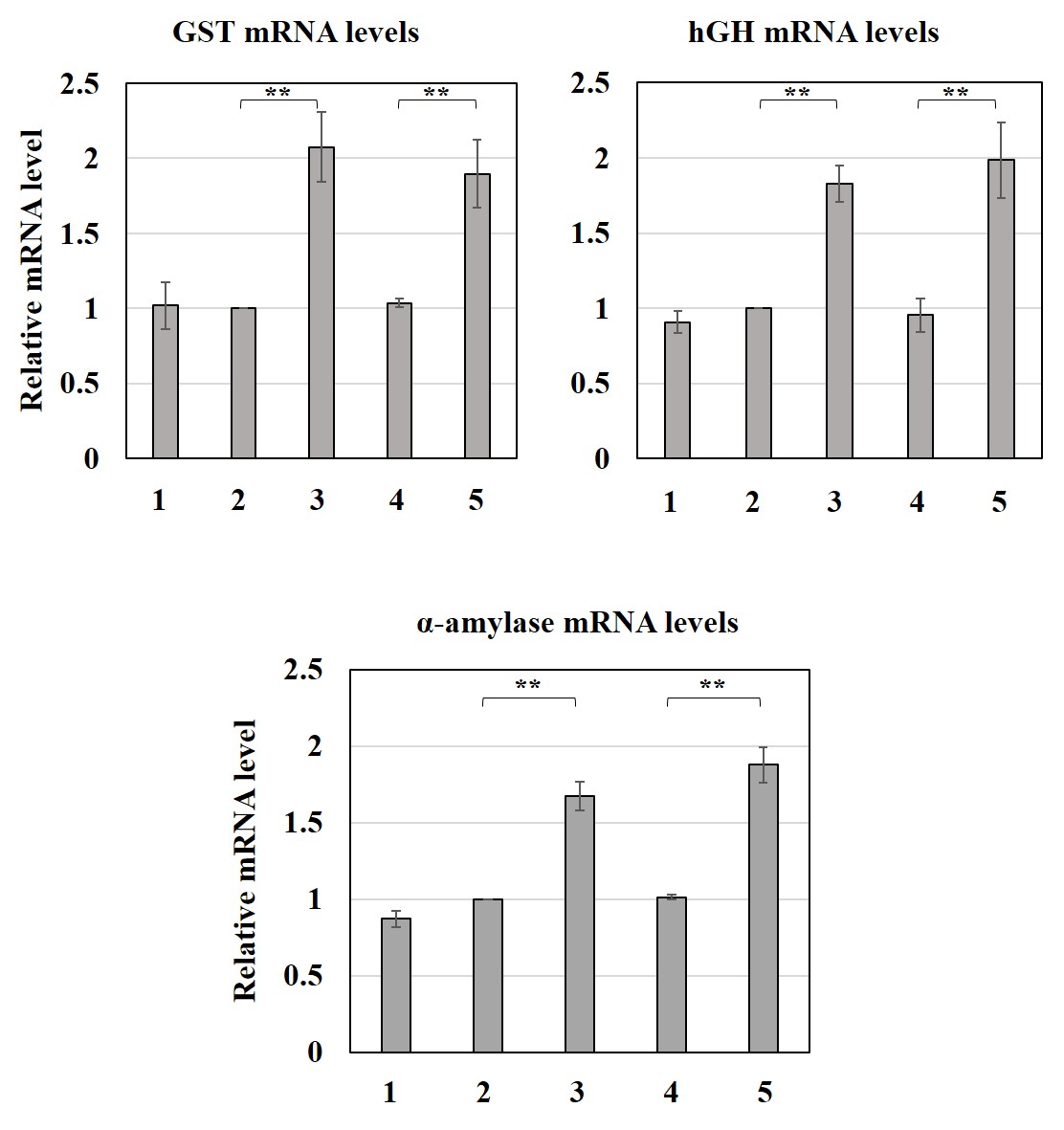


**Supplementary Figure S9.** Investigation of mRNA levels in the MCD and all BCDs harboring GST, hGH, and α-amylase. #1, pCB4270-GST, hGH, or α-amylase; #2, pCB4270B-GST, hGH, or α-amylase; #3, pCB4270V4B-GST, hGH, or α-amylase; #4, pCB4270BU-GST, hGH, or α-amylase; #5, pCB4270V4BU-GST, hGH, or α-amylase. All error bars represent the value of standard deviation which were calculated from three repeated experiments. ** p-value < 0.01 based on Student *t*-test.


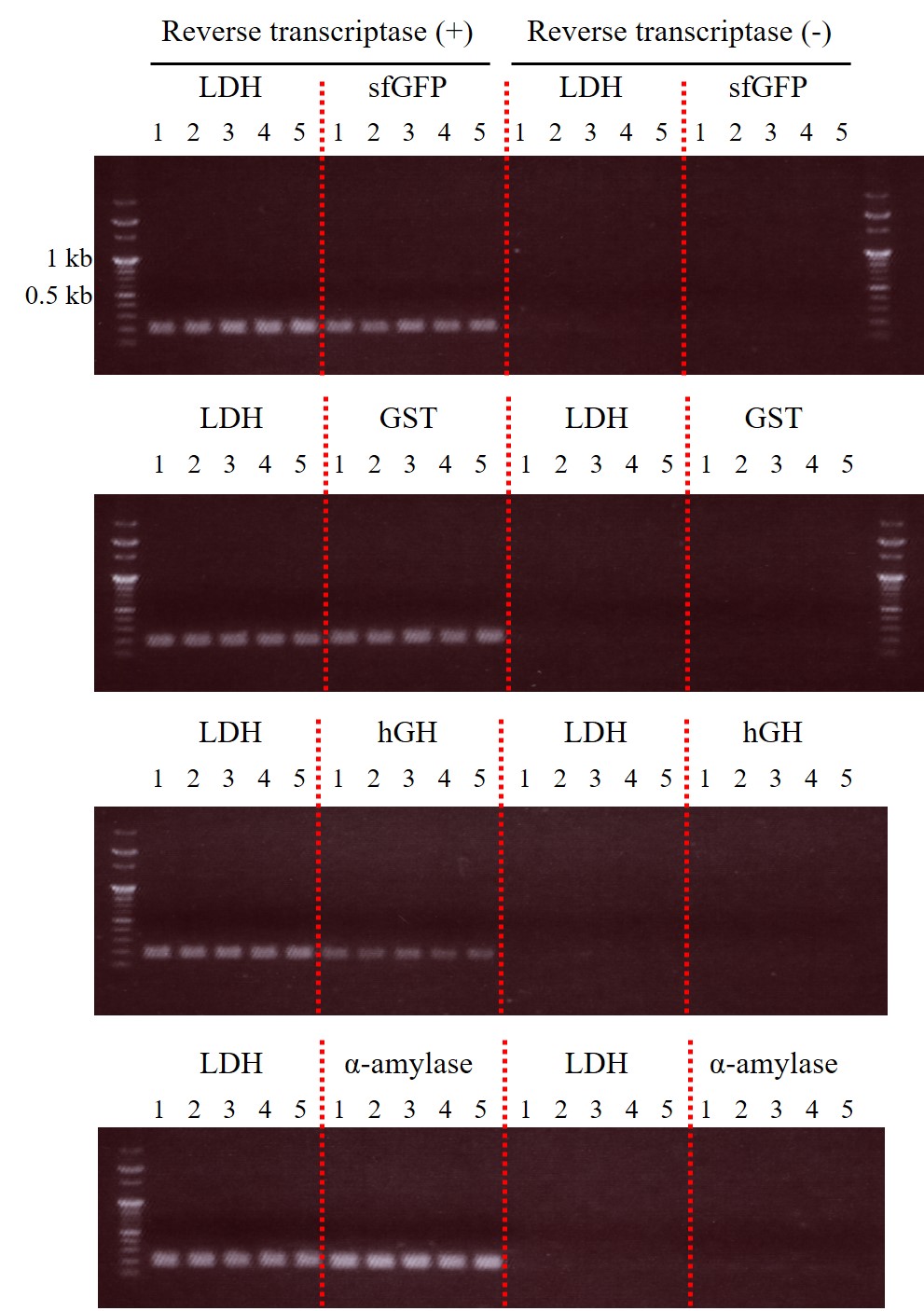


**Supplementary Figure S10.** RT-PCR of target genes in the MCD and all BCDs. #1, pCB4270-sfGFP, GST, hGH, or α-amylase; #2, pCB4270B-sfGFP, GST, hGH, or α-amylase; #3, pCB4270V4B-sfGFP, GST, hGH, or α-amylase; #4, pCB4270BU-sfGFP, GST, hGH, or α-amylase; #5, pCB4270V4BU-sfGFP, GST, hGH, or α-amylase.


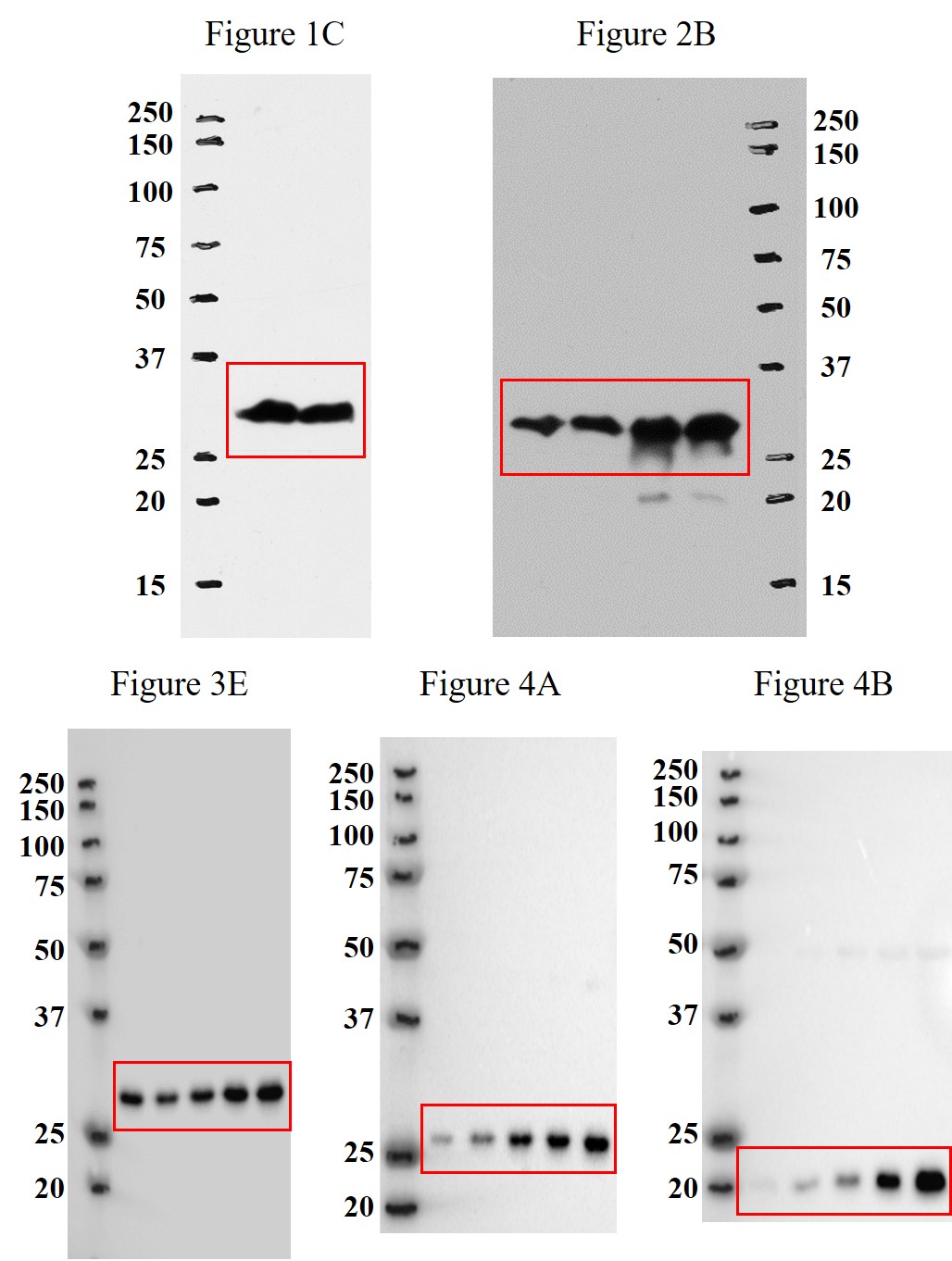


**Supplementary Figure S11.** Uncropped Western blots, shown in Figure 1C, 2B, 3E, 4A, and 4B.


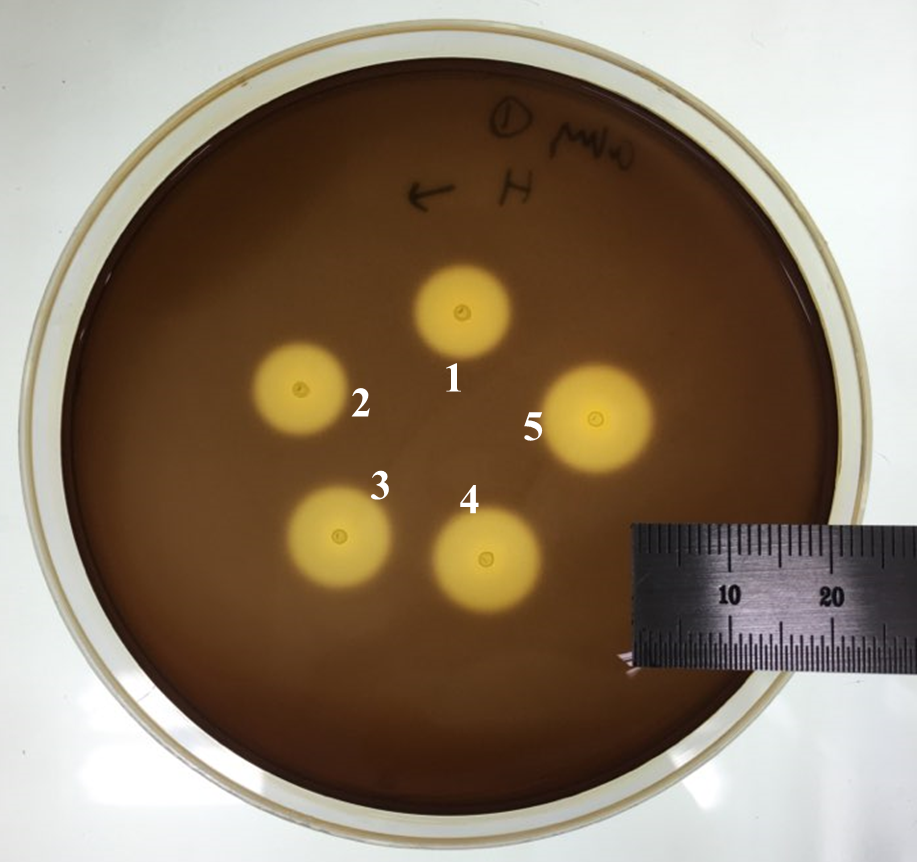


**Supplementary Figure S12.** Uncropped photograph of agar plate for amylase activity. Spots 1 to 5, *L. citreum* harboring pCB4270-amy, pCB4270B-amy, pCB4270V4B-amy, pCB4270BU-amy, and pCB4270V4BU-amy, respectively.
